# Functional diversity of nanohaloarchaea within xylan-degrading consortia

**DOI:** 10.3389/fmicb.2023.1182464

**Published:** 2023-05-31

**Authors:** Oleg Reva, Enzo Messina, Violetta La Cono, Francesca Crisafi, Francesco Smedile, Gina La Spada, Laura Marturano, Elena A. Selivanova, Manfred Rohde, Mart Krupovic, Michail M. Yakimov

**Affiliations:** ^1^Department of Biochemistry, Genetics and Microbiology, Centre for Bioinformatics and Computational Biology, University of Pretoria, Pretoria, South Africa; ^2^National Council of Research, CNR, Rome, Italy; ^3^Extreme Microbiology, Biotechnology and Astrobiology Group, Institute of Polar Research, ISP-CNR, Messina, Italy; ^4^Institute for Cellular and Intracellular Symbiosis, Ural Branch, Russian Academy of Sciences, Orenburg, Russia; ^5^Central Facility for Microbiology, Helmholtz Centre for Infection Research, Braunschweig, Germany; ^6^Archaeal Virology Unit, Institut Pasteur, Université Paris Cité, Paris, France

**Keywords:** nanohaloarchaeal-haloarchaeal symbioses, ecology of nanohaloarchaea, CRISPR, ncRNA, methylomics

## Abstract

Extremely halophilic representatives of the phylum *Candidatus* Nanohaloarchaeota (members of the DPANN superphyla) are obligately associated with extremely halophilic archaea of the phylum *Halobacteriota* (according to the GTDB taxonomy). Using culture-independent molecular techniques, their presence in various hypersaline ecosystems around the world has been confirmed over the past decade. However, the vast majority of nanohaloarchaea remain uncultivated, and thus their metabolic capabilities and ecophysiology are currently poorly understood. Using the (meta)genomic, transcriptomic, and DNA methylome platforms, the metabolism and functional prediction of the ecophysiology of two novel extremely halophilic symbiotic nanohaloarchaea (*Ca*. Nanohalococcus occultus and *Ca*. Nanohalovita haloferacivicina) stably cultivated in the laboratory as members of a xylose-degrading binary culture with a haloarchaeal host, *Haloferax lucentense*, was determined. Like all known DPANN superphylum nanoorganisms, these new sugar-fermenting nanohaloarchaea lack many fundamental biosynthetic repertoires, making them exclusively dependent on their respective host for survival. In addition, given the cultivability of the new nanohaloarchaea, we managed to discover many unique features in these new organisms that have never been observed in nano-sized archaea both within the phylum *Ca*. Nanohaloarchaeota and the entire superphylum DPANN. This includes the analysis of the expression of organism-specific non-coding regulatory (nc)RNAs (with an elucidation of their 2D-secondary structures) as well as profiling of DNA methylation. While some ncRNA molecules have been predicted with high confidence as RNAs of an archaeal signal recognition particle involved in delaying protein translation, others resemble the structure of ribosome-associated ncRNAs, although none belong to any known family. Moreover, the new nanohaloarchaea have very complex cellular defense mechanisms. In addition to the defense mechanism provided by the type II restriction-modification system, consisting of Dcm-like DNA methyltransferase and Mrr restriction endonuclease, *Ca*. Nanohalococcus encodes an active type I-D CRISPR/Cas system, containing 77 spacers divided into two loci. Despite their diminutive genomes and as part of their host interaction mechanism, the genomes of new nanohaloarchaea do encode giant surface proteins, and one of them (9,409 amino acids long) is the largest protein of any sequenced nanohaloarchaea and the largest protein ever discovered in cultivated archaea.

## Introduction

The last decade has revealed the astonishing diversity of ultrasmall archaea (< 500 nm in size, with cell volumes as low as 0.009 μm^3^) in nature, and the number of studies emphasizing their importance in the ecology, phylogeny, and evolutionary history of archaea is constantly growing. These nano-sized organisms belong to the tentative archaeal superphylum DPANN (an acronym of the names of the first described candidate phyla Diapherotrites, Parvarchaeota, Aenigmarchaeota, Nanoarchaeota, and Nanohaloarchaeota), which currently accommodates additional six candidate phyla (Huberarchaeota, Micrarchaeota, Mamarchaeota, Pacearchaeota, Undinarchaeota, and Woesearchaeota) (Castelle et al., 2018; Dombrowski et al., 2020). Members of DPANN are ubiquitous in nature and inhabit extremely diverse environments worldwide, including acid mine drainage (Golyshina et al., 2017) and highly alkaline sites (Vavourakis et al., 2016), brackish/freshwater, ocean sediments, hydrothermal vents, permafrost, and human microbiome (Dombrowski et al., 2019; for further references). The phylum “*Candidatus* Nanohaloarchaeota” is a representative extremely halophilic lineage within the DPANN superphylum. They inhabit various hypersaline ecosystems around the world: from natural salt lakes (Ghai et al., 2011; Narasingarao et al., 2012; Selivanova et al., 2018) and crystallizer ponds of solar salterns (La Cono et al., 2020; Leoni et al., 2020) to polyextreme habitats, such as salt crusts of the hyperarid deserts (Crits-Christoph et al., 2016; Finstad et al., 2017), Antarctic permanently cold hypersaline lakes (Hamm et al., 2019), and Eastern Mediterranean magnesium-saturated deep-sea brine lakes (La Cono et al., 2019). With cell volumes approaching the theoretical size limit for the unicellular form of life (0.008 μm^3^; Comolli et al., 2009) and genome sizes of approximately 1.0 Mbp, nanohaloarchaea are interesting in that they have multiple auxotrophies and so appear to depend, as ectosymbionts, on haloarchaeal hosts. However, as is true for all DPANN archaea, the most-basic questions regarding cellular metabolism and physiology, the nature of their trophic interaction with hosts, and their ecophysiological functions in the environment remain unanswered, as only a handful members of *Ca*. Nanohaloarchaeota have been grown and cultured under laboratory conditions in binary cultures. While an Antarctic haloarchaeon *Halorubrum lacusprofundi* is required for true parasitic growth of Antarctic nanohaloarchaea (proposed as *Ca*. Nanohaloarchaeum antarcticus) (Hamm et al., 2019), the nanohaloarchaeon *Ca*. Nanohalobium constans coexists with the chitinolytic haloarchaeon *Halomicrobium* sp. LC1Hm as a mutualistic ectosymbiont, an unprecedented finding (La Cono et al., 2020).

Here, we present a comparative study of metabolic functions, a repertoire of surface proteins likely involved in interaction with hosts, and the ecophysiology of two extremely halophilic nanohaloarchaea, *Ca*. Nanohalococcus occultus and *Ca*. Nanohalovita haloferacivicina. Our recent study to stably cultivate these nanohaloarchaea as members of xylan-degrading haloarchaeal consortia, obtained from brines and sediments of two distant hypersaline ecosystems (La Cono et al., 2023), has enabled the current study of their functional diversity that is key to understanding their ecology in nature. Metabolism, functional prediction of unique features, and both interaction with hosts and defense mechanisms were inferred from their cultivation experiments in binary cultures, whole genomes, transcriptomics, and DNA methylome analyses. Despite highly reduced, streamlined genomes and the absence of many fundamental biosynthetic repertoires, making them exclusively dependent on their respective host for survival, new nanohaloarchaeal strains have a complex set of genes (which occupy more than 10% of their entire genomes), involved in elaborated cell surface construction and adaptation to the ectosymbiotic lifestyle. Overall, they exhibit remarkable physiological and adaptive diversity that parallels what was recently described for other *Ca*. Nanohaloarchaeota members (Xie et al., 2022; Zhao et al., 2022). The present study greatly expands our understanding of the functional and genetic relationships between nanohaloarchaeal symbionts and their haloarchaeal hosts.

## Results and discussion

### General genome and defense mechanism characteristics of novel cultivated nanohaloarchaea

The genome of *Ca*. Nanohalococcus occultus SVXNc (SVXNc for short) consists of a single circular chromosome of 948,351 bp with molGC content of 47.4%. It contains single copies of 5S, 16S, and 23S rRNA genes located in three different loci, as well as 39 tRNA genes, 15 of which have an intron. Of the 1,046 protein-coding genes annotated in strain SVXNc, 620 (59.3% of the total) could be assigned to one of the NCBI Clusters of Orthologous Groups (COG) categories and 667 (63.8% of the total) to Archaeal Clusters of Orthologous Genes (arCOG). Notably, the 28,230 bp gene is present in this genome encoding for giant surface protein SVXNc_0300, similar to those found in genomes of other DPANN representatives, including nanohaloarchaea (see Hamm et al., 2019 for further references). The genome of *Ca*. Nanohalovita haloferacivicina BNXNv (BNXNv for short) consists of a single circular chromosome of 1,046,959 bp with molGC content of 45.0%. It contains single copies of 5S, 16S, and 23S rRNA genes located in three different loci, as well as 38 tRNA genes, four of which have an intron. Of the 1,170 protein-coding genes annotated in the strain BNXNv, 675 (57.7% of the total) could be assigned to one of the NCBI COG categories and 738 (63.1% of the total) to an arCOGs. Like *Ca*. Nanohalococcus occultus SVXNc, the large 26,643 bp gene encoding the giant surface protein was also found in the BNXNv genome and is discussed below.

**Figure 1:**
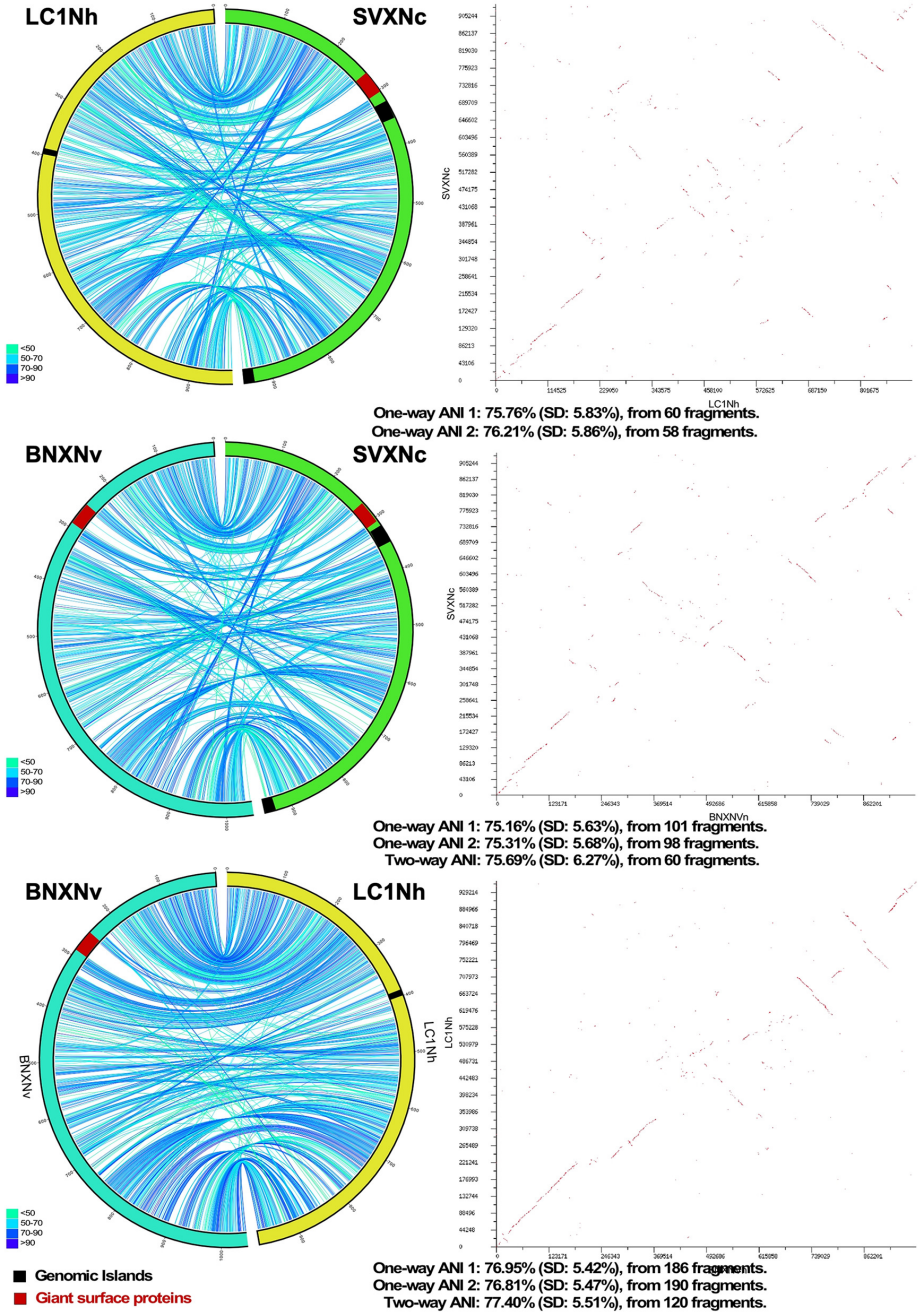
Pairwise genome comparison between *Ca*. Nanohalococcus occultus SVXNc, *Ca*. Nanohalovita haloferacivicina BNXNv, and *Ca*. Nanohalobium constant LC1Nh. Circos-based genome alignments and Dot-plot alignments are present in the left and right columns, respectively. Links indicate pairs of orthologous genes between the genomes, and the color is scaled to the percentage of amino acid identity levels (shown as the bottom-left inserts). The genomic islands predicted with IslandViewer4 are shown as black septa, while the positions of giant SPEARE proteins found in the BNXNv and SVXNc genomes are shown as red septa. For the SVXNc and LC1Nh couple, the similarity was too low for the two-way ANI index calculation, and only one-way ANI was shown.

Circos ribbon plots (Krzywinski et al., 2009) were used to visualize synteny between genomes of newly cultivated nanohaloarchaea and Ca. Nanohalobium constans LC1Nh (La Cono et al., 2020) used as a reference and to find clusters of orthologous genes. High overall collinearity and high similarity in terms of the gene context (>70% of amino acid identity) were found between all of them along with the presence of a few organism-specific genome rearrangements, such as the giant proteins and several genomic islands (Figure 1). These findings strongly suggest the common origin of all these nanohaloarchaea, even though they were isolated from geographically distinct sites and have different haloarchaeal hosts (La Cono et al., 2020, 2023).

**Figure 2:**
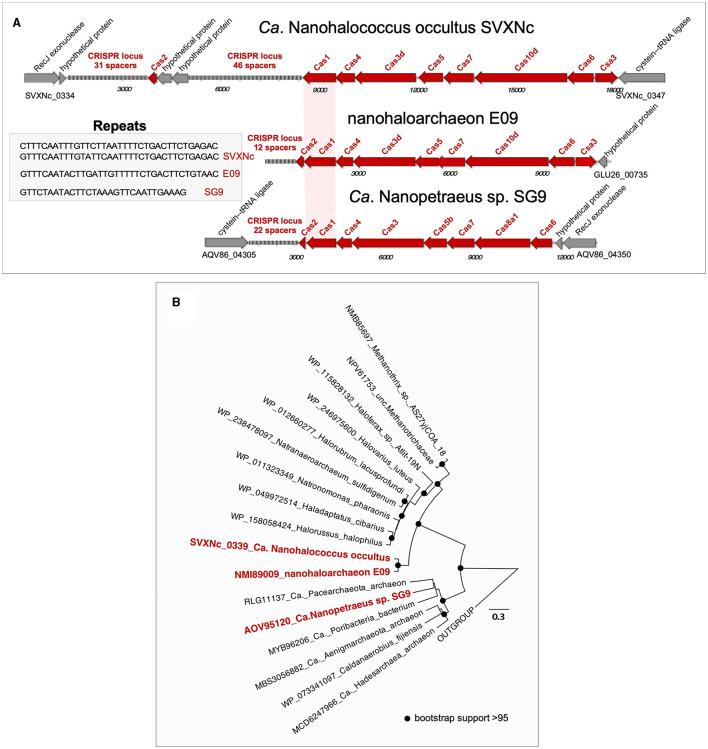
Structure of the CRISPR-Cas systems found in the *Ca*. Nanohalococcus occultus SVXNc genome and MAGs belonging to other two nanohaloarchaea **(A)** and phylogenetic analysis of Cas1 proteins **(B)**. The tree was constructed using the PhyML 3.0 plugin inside Geneious 7.1 with BLOSUM62 substitution model and 1,000 bootstrap replicates. Bootstrap values are shown as filled circles at the nodes.

Both genomic islands, found in the SVXNc genome, had a relatively lower GC content (41.5–44 mol%) compared to the 47.5 mol% calculated for the genome as a whole. A BLASTP search showed that these genomic islands may be acquired horizontally from other archaeal microorganisms. Noteworthy, no genomic islands were found within the *Ca*. Nanohalovita BNXNh genome, which is generally uncommon in nanohaloarchaea and other DPANN members. In the SVXNc genome, CRISPRFinder detected six potential CRISPRs regions (clusters of regularly interspaced short palindromic repeats), although a deeper analysis confirmed the presence of only one intact CRISPR/Cas system (SVXNc_0336-46) in the region from 328,855 bp to 342,986 bp, which corresponds to the location of one of the two genomic islands found. According to the CRISPR classification (Makarova et al., 2020), this system belongs to the type I-D CRISPR/Cas system and contains 77 spacers divided into two loci containing 31 and 46 spacers, respectively, and separated by the Cas2 (SVXNc_0336) gene and two genes encoding hypothetical proteins (SVXNc _0337-8) (Figure 2A). Apart from the MAG-assembled *Ca*. Nanopetraeus sp. SG9 (Crits-Christoph et al., 2016) and nanohaloarchaeon E09 (GCA_012927765) found in hypersaline salterns near Alicante (Spain) (Feng et al., 2021), we did not find evidence of other CRISPR/Cas systems in any other publicly available nanohaloarchaeal genomes. Remarkably, the 16,166 bp-long CRISPR region had a significantly lower G+C content (41.4 mol%) than the entire SVXNc genome (47.5%). To elucidate the origin of the CRISPR/Cas system in SVXNc, the *cas1* gene product was aligned with Cas1 proteins from various archaeal genomes. The resulting phylogeny showed that the SVXNc Cas1 protein, together with the E09 Cas1 protein, form a sister clade to Cas1 homologs from archaea of the classes *Methanomicrobia* and *Halobacteria* (Figure 2B). However, the genomes of *Halobacteria*, with the exception of that of *Haloquadratum walsbyi* (G+C % of 47.9) (Bolhuis et al., 2006), are characterized by high G+C% (>60 mol%), while the genomes of some extremely halophilic methanogens, such as *Methanohalarchaeum* and *Methanohalophilus*, have low G+C% (38 mol%) as does the CRISPR locus of SVXNc (Spring et al., 2010; Sorokin et al., 2017). These results support the potential origin of the nanohaloarchaeal CRISPR locus from halophilic methanogens.

Although the role of CRISPR/Cas systems as protective agents against viruses and other potential mobile agents that can threaten the integrity of the genome is well known, the analysis of spacer sequences as records of possible past encounters is still very challenging. To date, many haloviruses have been identified that attack members of halophilic microbial communities (see Atanasova et al., 2015; Truitt and Deole, 2021 for further references). However, we did not find any similarity between the SVXNc spacers and the known halovirus sequences, suggesting that this nanohaloarchaeon has dealt with or encountered unknown viruses or other mobile genetic elements in the past. Alternatively, given that *Ca*. Nanohalococcus SVXNc, which has a very limited anabolism, appears to obtain the nucleotides required for the growth by the uptake of DNA from the environmental (eDNA), an attractive possibility is that its CRISPR/Cas system participates in the degradation of the imported eDNA, which has recently been proposed for other nano-sized prokaryotes, namely members of proposed new order *Nucleotidisoterales* within *Ca*. Nanohaloarchaeota (Xie et al., 2022) and bacterial “vampires” of the Gracilibacteria lineage within Candidate Phyla Radiation (Moreira et al., 2021; Yakimov et al., 2022). In this context, and as if to confirm this assumption, a direct BLASTn search (Altschul et al., 1997) was carried out against the entire collection of nr/nt nucleotides in the NCBI database (August 10, 2022) using the more dissimilar sequences (discontiguous megablast) option. Only one 91.11% identity match, namely the spacer 39 against a *Haloplanus* sp. GDY1 plasmid (CP098517), was found.

**Figure 3:**
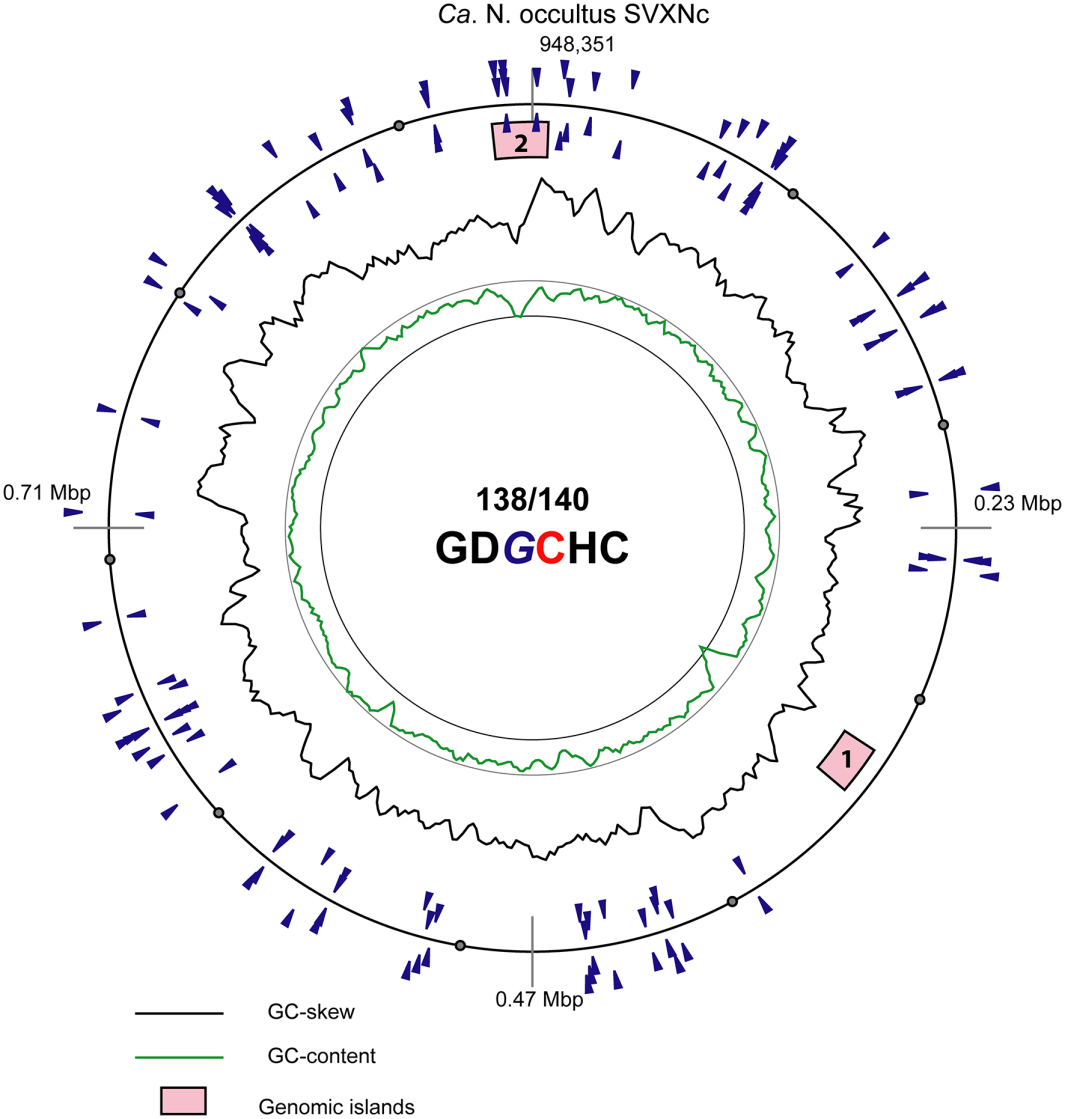
Atlas view of the circular chromosome of the strain SVXNc. Histogram graphs show GC-skew and GC-composition fluctuations in a 5,000 bp sliding window. Locations of motifs GDGCHC with bipartite methylated cytosine residues on the direct and reverse complement DNA strands are depicted by blue triangles outside and inside of the outermost ring representing the chromosome. In the motif sequence GDGCHC, the methylated cytosine (C) is shown red, and the guanine residues (G) opposing the methylated cytosine on the complementary strand are italicized. Locations of the two identified genomic islands are depicted by numbered pink boxes.

In addition to CRISPR/Cas system, *Ca*. Nanohalococcus SVXNc encodes another defense mechanism courtesy of the type II restriction-modification (RM) system, consisting of a Dcm-like DNA methyltransferase (SVXNc_0752) and a restriction endonuclease Mrr (SVXNc_0757). These genes do not form an operon, and both are expressed in the SVXNc transcriptome (Supplementary Table S1). Bipartite methylation of cytosine residues at GD*G***C**HC type II recognition sites was confirmed for *Ca*. Nanohalococcus SVXNc by PacBio sequencing of the genome in three repeats (Figure 3, the methylated cytosine residue is shown in bold and highlighted by color; the guanine residue opposing the methylated cytosine on the complementary strand is italicized). Out of 140 palindromic motifs GDGCHC found in the genome on both DNA strands, 138 motifs were methylated at both DNA strands (Supplementary Table S2). Only one site, GTGCAC, located at the 576132-576137 region within the *ppsA* coding sequence was not methylated on either strand in all three repeats of sequencing. Either this site was somehow protected from methylation, or it could be that this sequence had not been recognized by the methyltransferase as it was the only GTGCAC sequence in the whole genome of *Ca*. Nanohalococcus. Methylation of all other recognition motifs in the genome suggested that this restriction-modification system was fully functional and could protect the genome from phage invaders. The BNXNh nanohaloarchaeon, in turn, has another, the type III RM system, consisting of Mod DNA methyltransferase (BNXNv_0801) and Res restriction endonuclease (BNXNv_0802), co-located in the same operon. For this nanohaloarchaeon, methylation of adenine residues can be expected. To our knowledge, these are the first indications of experimentally confirmed DNA methylation patterns and an active RM system across the entire DPANN superphylum.

### Reconstruction of the core metabolism of novel cultivated nanohaloarchaea

*Protein translocation systems, membrane-associated proteases, and transporters*. An overview of the metabolic potential and the core metabolism is illustrated in Supplementary Figure S1. Among the totals of 1,088 and 1,199 proteins predicted from the SVXNc and BNXNv genomes, respectively, some were annotated by BlastKOALA (Kanehisa et al., 2016) as putative members of the “Membrane Transport” category. Apparently, both organisms possess the general secretion system Sec as the major means of protein secretion, which includes the signal recognition particle complex (SRP), and six different genes belonging to this protein-trafficking system were recognized: SecD (SVXNc_1062 and BNXNv_1183), SecF (SVXNc_1063 and BNXNv_1184), SecG (SVXNc_0958 and BNXNv_1035), SecY (SVXNc_0611,0894 and BNXNv_1117), Sss1 (SVXNc_0114 and BNXNv_0098), the SRP receptor FtsY (SVXNc_0393 and BNXNv_0691), and the targeting protein Ffh (SVXNc_0398 and BNXNv_0686) as well as the gene for non-coding SRP RNA. The twin-arginine translocation (Tat) system, that exports folded proteins from cells and is represented by a single TatC protein in the SVXNc genome (SVXNc_0793) only, and thus, is likely inactive in both nanohaloarchaea or this gene has another function. Signal peptidases of the SppA type (SVXNc_0702 and BNXNv_0859) and archaeal type I (SVXNc_0770 and BNXNv_0034,0327,0746) are the principal intra-membrane peptidases responsible for processing the majority of exported proteins. Two rhomboid-family proteases (SVXNc_0214 and BNXNv_0161) are the intra-membrane serine proteases that cleave other proteins, including S-layer glycoproteins (SVXNc_0478,0977 and BNXNv_0277,0530,0625,0988) within their transmembrane domains.

A total of 11 and 12 genes were annotated as components of ABC-type transporter systems in the SVXNc and BNXNv genomes, respectively. Among them, there are genes encoding a putative peptide ABC transport system SalXY (SVXNc_0976,0978-9 and BNXNv_0529,0531-32) and three uncharacterized ABC-2 type transporter complexes. Sugars can be imported into the cytoplasm either by one of these uncharacterized ABC-type transporters, putative permeases PerM (SVXNc_0693 and BNXNv_0869,0978), and/or by major facilitator superfamily (MFS) permeases found in the genomes. Notably, the SVXNc genome harbors only one gene of MFS permease (SVXNc_0852) (similar to *Ca*. Nanohalobium constans), whereas six MFS-coding genes (BNXNv_0572,0893,0955,0995-6,1020) were found in the BNXNv genome. As we mentioned previously (La Cono et al., 2020), this putative transporter system seems to be specific to nanohaloarchaea since we failed to find any close homologs (>25% of aa sequence identity) with annotated functions.

Transport of amino acids and citrate(isocitrate) into the cells of the strains SVXNc and BNXNv likely occurs *via* amino acid permeases PerM (SVXNc_0693 and BNXNv_0869) and tripartite tricarboxylate transporters (SVXNc_1057-8 and BNXNv_1180-1), respectively. The phosphate/sulfate Pit permeases (SVXNc_0158,0539 and BNXNv_0146,0553) likely participate in the transportation of inorganic ions, osmotic homeostasis, and resistance to heavy metals along with K^+^-dependent mechano-sensitive MscS channels (SVXNc_0010,0482,0867 and BNXNv_0365,0395,0547,0911), K^+^-dependent Na^+^-Ca^2+^ ECM27 exchanger (SVXNc_0053 and BNXNv_0458), Na^+^/K^+^: H^+^ antiporter of Kef type (SVXNc_0165-6,0900 and BNXNv_0923-4,0968), NADH-dependent K^+^ transport system of Trk type (SVXNc_0862-3 and BNXNv_0906-7), zinc/iron permease of the ZIP family (SVXNc_0931 and BNXNv_0999), Fe^2+^/Mn^2+^ transporters MgtA and CCC1 (SVXNc_0063,0637 and BNXNv_0226,0424,1013), an iron complex transporter FepB (BNXNv_0613, found in the BNXNv genome only), and P-type heavy metal (cations)-transporting ATPases (SVXNc_0063,0584,0689,0931,1046 and BNXNv_0038,0226,0242,0251,0499,0999,1013). Apart from this transporter machinery, the *Ca*. Nanohalococcus occultus SVXNc genome harbors a fully operational multicomponent sodium/proton antiporter MnhA-G system (SVXNc_0517-23) that utilizes the proton motive force to expel intracellular sodium ions to the extracellular milieux. As revealed by a BLAST search, out of all members of the DPANN superphylum, the Mnh antiporter system was found in only four nanohaloarchaeal genomes. Phylogenetic analysis confirmed that this system was apparently acquired by horizontal gene transfer from haloarchaea.

#### Energy production and catabolism

The genomes of the strains SVXNc and BNXNv contain no genes associated with the carbon-fixation pathways, pointing toward their strict heterotrophic lifestyle. Both genomes lacked pivotal enzymes needed for the pentose phosphate pathway and the ribulose monophosphate pathway, so these microbes are incapable of metabolizing pentose sugars. Given that they also lack genes encoding most components of the tricarboxylic acid cycle and any of the respiratory complexes (NADH dehydrogenase complex, functional cytochrome oxidases, and terminal reductases), we infer a strictly anaerobic fermentation-based lifestyle. Membrane-bound proton-translocating pyrophosphatases were also not found, so the maintenance of the chemiosmotic membrane potential, osmotic stress response, and pH homeostasis should fully rely on an archaeal-type H^+^-ATPase (SVXNc_0673,0675-82 and BNXNv_0873-81), Kef-type K^+^/H^+^ antiporters (SVXNc_0165-6,0900 and BNXNv_0924,0968), seven-subunit Mnh-type Na^+^/H^+^ secondary antiporter (SVXNc_0517-23, found only in the SVXNc genome), and possibly several other unidentified systems functioning as proton-translocating membrane pumps.

A complete set of genes of the archaeal version of the dissimilative Embden–Meyerhof–Parnas (EMP) pathway of glycolysis was identified in both genomes. None of the genes of the Entner–Doudoroff pathway were found in any of the oxidative and non-oxidative variants of the pentose phosphate pathway (Supplementary Figure S1). Thus, in the absence of membrane respiratory complexes, the EMP pathway is the only way of gaining energy by fermentation, namely by substrate-level phosphorylation. As it was demonstrated recently (La Cono et al., 2020), the EMP pathway of energy production with some variations at the upstream steps of glucose metabolism exists in all sequenced nanohaloarchaea and also in some halophilic methanogenic archaea but it was missed in haloarchaea (Gonzalez-Ordenes et al., 2018). Employing ADP as the phosphoryl donor, the phosphorylation of both glucose and fructose-6-phosphate is likely catalyzed by only one enzyme, the bifunctional ADP-dependent phosphofructokinase/glucokinase PfkC (SVXNc_0128 and BNXNv_0111) of the ribokinase family (EC 2.7.1.146; EC 2.7.1.147). BNXNv has an additional phosphofructokinase FruK (BNXNv_0774) to phosphorylate fructose-6-phosphate. Fructose-1,6-biphosphate is further converted *via* a fructose-biphosphate aldolase FbaB (SVXNc_0147 and BNXNv_0133) to dihydroxyacetone phosphate and glyceraldehyde-3-phosphate (GAP). These intermediates enter the lower portion of the EMP pathway and are transformed by G?pA dehydrogenase (SVXNc_0140 and BNXNv_0123), phosphoglycerate kinase Pgk (SVXNc_0217 and BNXNv_0164), phosphoglycerate mutase Gmpl (SVXNc_1002 and BNXNv_1066), and enolase Eno (SVXNc_0290 and BNXNv_0311) ending up with phosphoenolpyruvate (PEP). The final step of the glycolysis consisting of the conversion of PEP to pyruvate can be catalyzed by a pyruvate kinase PykF (SVXNc_0488 and BNXNv_0446) and two AMP/Pi-dependent phosphoenolpyruvate synthases/pyruvate water dikinases PpsA (SVXNc_0254,0565,0650 and BNXNv_0201,1153). The synthesis of pyruvate from PEP by the water dikinase is energetically more favorable than by the pyruvate kinase since it can directly synthesize ATP from phosphate and AMP, produced by the ADP-dependent PfkC (Falb et al., 2008; Atomi and Reeve, 2019; La Cono et al., 2020). As mentioned above, the genomes of SVXNc and BNXNv encode the complete archaeal-type H^+^-ATPase complex (SVXNc_0673,0675-82 and BNXNv_0873-81). Thus, in addition to many important homeostatic and anabolic reactions, the energy generated during the glycolysis can be used to maintain the cytoplasmic pH within a biocompatible range through the F_0_ rotor of the A-type ATPase, resembling the metabolism of other strictly fermentative organisms that lack the electron transport chains and are incapable of respiration.

As in many DPANN organisms, the novel nanohaloarchaea have many genes designated for pyruvate metabolism, which include a pyruvate dehydrogenase complex (PDH) consisting of AcoAB, AceF, and Lpd enzymes (SVXNc_0064-67,0758 and BNXNv_0048-51,0239), lactate dehydrogenase LdhA (SVXNc_0992 and BNXNv_0542), NAD-dependent malic enzyme SfcA (SVXNc_0069 and BNXNv_0060), and a short-chain alcohol dehydrogenase FabG (SVXNc_1061 and BNXNv_0428). The PDH complex decarboxylates pyruvate to acetyl-CoA. An ADP-forming acetyl-CoA synthetase AcdA (SVXNc_0068 and BNXNv_0052) likely completes the oxidative pathway of pyruvate metabolism leading to the formation of ATP and acetate. The presence of the other two pyruvate-transforming enzymes indicates that this metabolite could also be used in several reductive pathways using pyruvate as an electron acceptor in its conversion to malate and ethanol possibly to reduce the excess of NADH formed during pyruvate oxidation. It is noteworthy that all these enzymes of pyruvate metabolism were highly expressed in the nanohaloarchaeal strain SVXNc (Supplementary Table S1).

The genes of the EMP pathway in the SVXNc and BNXNv genomes can be used also in the gluconeogenetic pathway due to the presence of a fructose-1,6-bisphosphatase Fbp (SVXNc_0148 and BNXNv_0134) that switches between the glycogenesis and the gluconeogenesis. The strains SVXNc and BNXNv have enzymes necessary for the synthesis of glycogen: a phosphomannomutase/phosphoglucomutase ManB (SVXNc_0127,0558 and BNXNv_0110,1160), nucleotidyltransferase (SVXNc_0671 and BNXNv_0476,0884), glycogen synthase RfaG (SVXNc_0131 and BNXNv_0114), and a glycogenin-like protein BNXNv_1210 found only in BNXNv. This finding supports the hypothesis that both these nanohaloarchaea can perform a *de novo* synthesis of glucose and further convert it for intracellular storage in the form of glycogen, a process proposed as a hallmark not only of all sequenced members of *Ca*. Nanohaloarchaeota but also of most other DPANN organisms (Castelle et al., 2015, 2018; Dombrowski et al., 2019). Remarkably, this mode of carbon storage is absent in all known members of haloarchaea (La Cono et al., 2020). In accordance with the ability to synthesize glycogen, both nanohaloarchaea strains used in this study were also capable of the decomposition of glycogen into glucose. This process involves a glycogen debranching enzyme GDB1 (SVXNc_0130 and BNXNv_0113) and various alpha-glucan hydrolases: alpha-amylase of the glycosyl hydrolase (GH) 57 family (SVXNc_0138 and BNXNv_0120), alpha-amylase of the GH13 family (BNXNv_0413, found only in the BNXNv genome), and a glucoamylase of the GH15 family (SVXNc_0760, found only in the SVXNc genome). All the enzymes involved in the metabolism of pyruvate and synthesis/decomposition of glycogen were actively expressed in Nanohalococcus occultus SVXNc as revealed by the transcriptome analysis (Supplementary Table S1). This suggests a highly dynamic turnover of glucose and the storage compounds in this organism.

#### ROS sensing and redox homeostasis

Redox homeostasis is important for all living organisms. In the absence of any of the respiratory complexes, both strains of nanohaloarchaea must stick to a strictly anaerobic fermentative lifestyle. However, they are obligatory-dependent on their aerobic host *Haloferax lucertense* (La Cono et al., 2023). Inhabiting aerobic environments requires an extended tolerance to oxidative stress. In both these archaeal nanoorganisms, thioredoxin most likely plays the most important role in the maintenance of redox homeostasis and in catalyzing various biochemical redox reactions controlled by a thiol-disulfide isomerase TrxA (SVXNc_0146,0837 and BNXNv_0132,0394,0617), thioredoxin reductase TrxB (SVXNc_0516 and BNXNv_0612), ribonucleotide reductase NrdA (SVXNc_0533 and BNXNv_0637), peptide methionine sulfoxide reductase MsrA (SVXNc_0607,0994 and BNXNv_0501,0935,1059), and a peroxiredoxin Bcp (SVXNc_0159,0582 and BNXNv_0618). The thioredoxin system is involved in peroxide removal by a superoxide dismutase SodA (SVXNc_0514 and BNXNv_0610). Both strains have an additional alkyl hydroperoxide reductase AhpF (SVXNc_0616 and BNXNv_0455) that confers a more active peroxide scavenging during oxidative stresses (Kashima and Ishikawa, 2003). Other important mechanisms of redox homeostasis involve the glutathione (GSH)-dependent systems (Zhang and Forman, 2012). GSH is synthesized by glutamate–cysteine ligase and glutathione synthase GshAB (SVXNc_0869-70 and BNXNv_0913,0915). GSH serves as an oxidizing substrate in reactions controlled by glutaredoxin GrxC (SVXNc_0734 and BNXNv_0819). GSH may play an important role in the removal of toxic methylglyoxal compounds resulting from the isomerization of several glycolysis intermediates to avert oxidation stress (Rawat and Maupin-Furlow, 2020). This process is controlled by a GSH-dependent lactoylglutathione lyase GloA (SVXNc_0045 and BNXNv_0036). The strain SVXNc has an additional hydroxyacylglutathione hydrolase SVXNc_0657 that presumably controls a similar reaction of conversion of methylglyoxal compounds to (R)-S-lactoylglutathione with a further degradation to lactate. All the abovementioned genes are expressed in SVXNc (Supplementary Table S1).

### The limited anabolic capability of the cultivated nanohaloarchaea

The characteristic genomic features of *Ca*. Nanohalococcus occultus SVXNc and *Ca*. Nanohalovita haloferacivicina BNXNv are consistent with the predictions made before for uncultured and cultured nanohaloarchaea and also for other DPANN organisms (Castelle et al., 2015, 2018; Dombrowski et al., 2019; Hamm et al., 2019; La Cono et al., 2020). Despite the presence of a full set of genes for chromosome maintenance and replication, a significant genome reduction was observed, making these nanoorganisms unable to synthesize the most necessary metabolic precursors including the majority of amino acids and lipids, nucleotides, and cofactors. Indeed, the enzymatic suite of *de novo* biosynthesis of amino acids is limited in both strains (Supplementary Figure S1) with only a few genes present including an asparagine synthase AsnB (SVXNc_0203 and BNXNv_0940) and a threonine dehydratase Tdh (SVXNc_0578), found only in the SVXNc genome. BNXNv has an operon of genes involved in aromatic acid biosynthesis that includes two prephenate dehydratases PheA and TyrA (BNXNv_0067,69), aspartate/tyrosine/aromatic acid aminotransferase AspB (BNXNv_0068), and a chorismate mutase (BNXNv_0070). Both nanohaloarchaea have an amino acid transporter PotE of the APC permease family (SVXNc_0483 and BNXNv_0526) that likely takes part in the uptake of aromatic amino acids from the extracellular environment. Remarkably, the phylogenetic analysis of the PotE revealed that in addition to nanohaloarchaeon M3_22 (Feng et al., 2021), all other DPANN members lack this type of transporter suggesting that this permease was likely acquired by horizontal gene transfer from haloarchaea.

Regarding the metabolism of purine and pyrimidine, we identified only a few genes encoding various kinases involved in the inter-conversion of nucleoside phosphates, adenylate kinase, thymidylate synthases, kinases, and thymidine kinases, enzymes involved in the downstream stages of nucleotides synthesis or nucleotide salvage (Supplementary Figure S1). Similarly, the capability for *de novo* synthesis of cofactors is virtually absent, except for several genes involved in completing the synthesis and maturation of the most-common cofactors. For example, both nanohaloarchaea have a molybdopterin-synthase MoeB (SVXNc_0838 and BNXNv_0396). Molybdopterin is an enzymatic cofactor of several archaeal oxidases and a component of the tungsten cofactor that catalyzes ferredoxin-dependent oxidoreductases oxidizing aldehydes, formaldehyde, and glyceraldehyde-3-phosphate (Buessecker et al., 2022). Pterin-4a-carbinolamine dehydratase PhhB (SVXNc_0711 and BNXNv_0848) synthesizes tetrahydrobiopterin that is another important cofactor of amino acid metabolism. The final step of the synthesis of the redox cofactor NAD^+^ is controlled by a nicotinamide mononucleotide adenylyltransferase NadR (SVXNc_0143 and BNXNv_0126), which was found in both genomes. In addition to this gene, the BNXNv genome has a phosphoribosyltransferase/nicotinamidase PncAB (BNXNv_0901-2) contributing to additional steps of NAD^+^ biosynthesis from nicotinate. Both genomes harbor a thymidylate synthase ThyA (SVXNc_0152 and BNXNv_0141) and a glycine/serine hydroxymethyltransferase GlyA (SVXNc_0154 and BNXNv_0142) involved in the turnover of tetrahydrofolate derivatives. However, this system is more developed in the SVXNc genome due to the presence of a dihydrofolate reductase FolA (SVXNc_0151) and a methylenetetrahydrofolate dehydrogenase FolD (SVXNc_1072), which are absent in the BNXNv genome.

Tetrahydrofolate derivatives are the donors or acceptors of C_1_-groups in many biosynthetic pathways, particularly in the synthesis of S-adenosyl-methionine (SAM) and in serine-glycine turnover. The SAM synthesis requires cobalamin as a cofactor, and the genomes of both strains have a PduO-based adenosylcobalamin salvage system (SVXNc_0941 and BNXNv_1010); however, an archaeal SAM-synthetase MetK was found only in the BNXNv genome (BNXNv_0766). SAM is the universal donor of methyl groups used by the two SAM-dependent methyltransferases found in the SVXNc and four such genes found in the BNXNv genomes (SVXNc_0183,1081 and BNXNv_0119,0321,0522,0971). One important SAM-based biosynthetic pathway shared by both nanohaloarchaea is the synthesis of diphthine controlled by DPH5 diphthine synthase/diphthamide methyltransferases (SVXNc_1032 and BNXNv_1096). Diphthine is a compound found in archaeal and eukaryotic cells that is indispensable for the functionality of the elongation factor 2 (Su et al., 2013). SAM also provides with methyl groups in the DNA restriction-modification systems, found in both microorganisms.

As was already mentioned when analyzing the *Ca*. Nanohalobium constans LC1Nh genome (La Cono et al., 2020), a caveat in this and other reconstructions are that more than one-third of the proteins encoded in the SVXNc and BNXNv genomes (35.9 and 37.0%, respectively) were annotated as hypothetical proteins and could not be assigned to any functional category. This raises the question of whether these organisms might encode some novel enzymes that drive either known or yet unknown metabolic pathways.

### Cell-surface structures of the cultivated nanohaloarchaea and their predicted role in the interaction with the host

In sharp contrast to their simplified core metabolism and limited anabolic capabilities, the nanohaloarchaeal strains SVXNc and BNXNv possess a sophisticated set of genes related to the construction of an elaborated cell surface, which seems to be a common hallmark of many DPANN archaea (Huber et al., 2002; Jahn et al., 2008; Golyshina et al., 2017; Jarett et al., 2018; Hamm et al., 2019; La Cono et al., 2020). The SVXNc and BNXNv genomes encode various glycosyl transferases (11 and 12 genes, respectively) which belong to the GT1, GT2, and GT8 families. According to transcriptomic data, the majority of them are expressed in the SVXNc cells (Supplementary Table S1). This indicates that this nanohaloarchaeon expends substantial amounts of energy on the biosynthesis of precursors for glycosylation processes and the synthesis of polysaccharides and glycoproteins as principal components of the extracellular matrix, including N-glycosylated S-layer proteins (SVXNc_0478,0977 and BNXNv_0277,0530,0625,0988) and many other predicted surface proteins.

The BNXNv genome contains an operon comprising genes of phytoene dehydrogenase CrtD, squalene synthetase CrtB, and geranylgeranyl pyrophosphate synthase IsdA (BNXNv_0461-3). Using alkyldecaprenyl pyrophosphate as a substrate, these enzymes might participate in the synthesis of extracellular squalene-containing lipid monolayer, similar to that found in many extremely halo(natrono)philic archaea (Gilmore et al., 2013). None of the known homologs of these genes were found in the SVXNc genome and only genomes of three uncultured nanohaloarchaea, namely *Ca*. Nanosalina (Narasingarao et al., 2012) and two unclassified strains, recovered from salterns near Alicante (Spain) (Feng et al., 2021), had these genes. The SVXNc and BNXNv genomes have 15 and 10 genes, respectively, likely involved in the synthesis and assembly of archaella and filament proteins. Four of these genes (SVXNc_1016-7 and BNXNv_1081-2) were situated in large operons (SVXNc_1003-31 and BNXNv_1067-93) that host up to 22 hypothetical proteins resembling the structure of euryarchaeal archaella operons (Albers and Jarrell, 2018). It remains to be determined whether these flagellar structures are used for motility of detached SVXNc and BNXNv cells, or whether they facilitate attachment of these archaea to the host cells.

**Figure 4:**
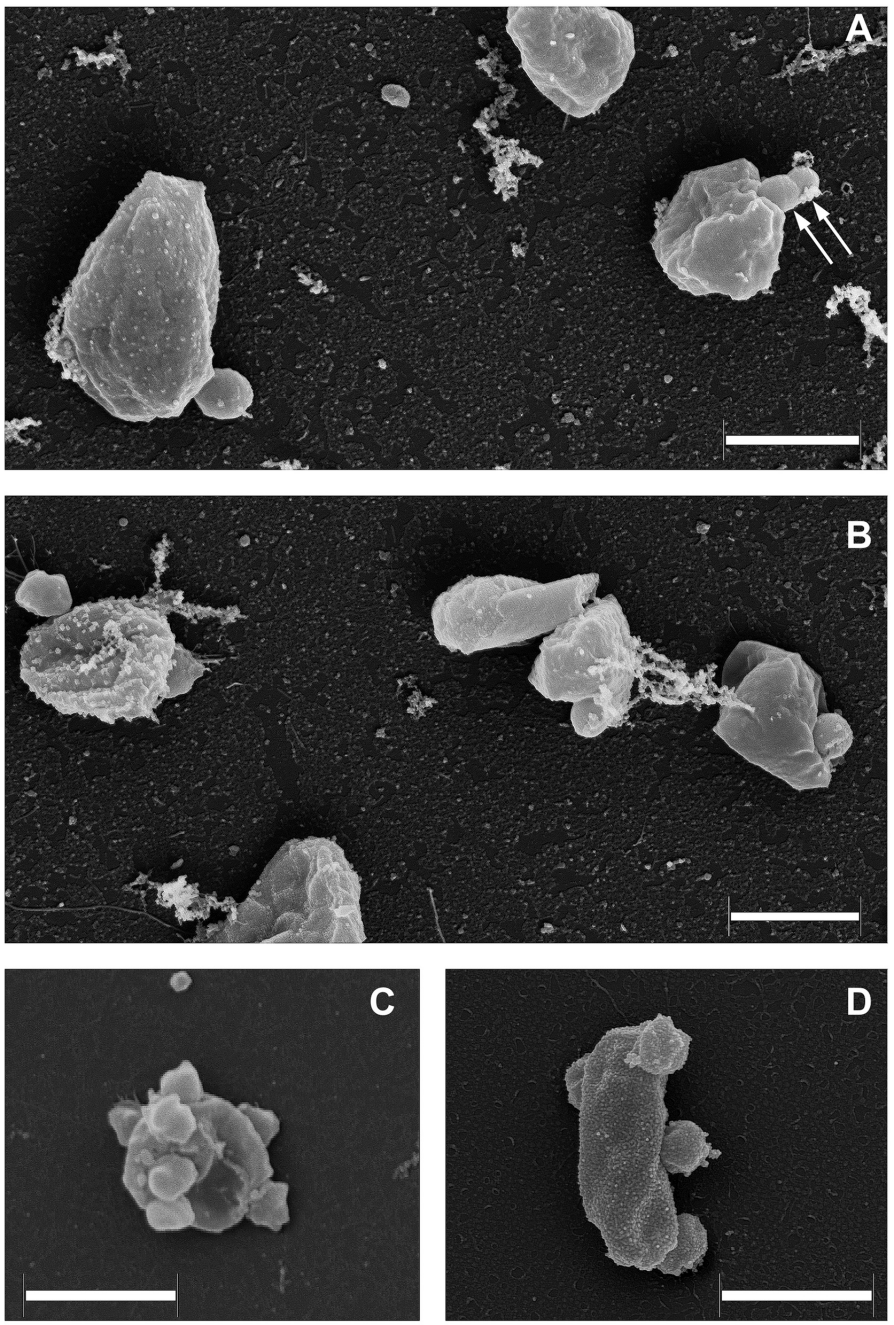
Field emission scanning electron micrographs of *Ca*. Nanohalovita haloferacivicina BNXNv **(A, B)** and *Ca*. Nanohalococcus occultus SVXNc **(C, D)** attached to their respective hosts, *Haloferax lucertense* BNX82 and SVX82. The images show a strong difference in the median multiplicity of host-attached nanosymbionts: *Ca*. Nanohalovita BNXNv−1–2 cells/host cell; *Ca*. Nanohalococcus SVXNc−4–7 cells/host cell. Dividing BNXNv cells are indicated by white arrows **(A)**. Scale bars represent 1,000 nm.

As it was demonstrated recently (La Cono et al., 2023), both transmission and scanning electron microscopy revealed the spatial distribution of nanohaloarchaeal cells on a single *Haloferax lucentense* host cell, suggesting a strong intercellular interaction (Figure 4). Given this, we looked for genomic evidence of putative mediators of these cross-species cellular interactions. At least six strain-specific secreted or membrane-bound surface proteins, containing either a polycystic kidney disease (PKD) domain (SVXNc_0035) or multiple (up to five) concanavalin A-like/lectin laminin (LamG) domains (SVXNc_0801,0806,0807,0813,0831), were found in the *SVXNc* genome. The surface proteins containing these domains could serve a variety of purposes by mediating interactions with carbohydrates or glycosylated proteins, and they were also predicted to be involved in the specific recognition and fixation of DPANN organisms to their hosts (Castelle et al., 2015, 2018; Golyshina et al., 2017). Remarkably, the genes encoding three of the LamG domain-containing proteins are organized in one operon together with the genes of 1,693 amino acids long trimeric autotransporter adhesin-like protein (SVXNc_0803) and 1,386 amino acids long protein, containing Microbial Surface Components Recognizing Adhesive Matrix Molecules (MSCRAMM) clumping factor A domain and knob domain of R-type pyocins (SVXNc_0798). MSCRAMM and knob-like domains occur in host-recognition and binding proteins, where the knob-like domain may interact with sialic acid (Buth et al., 2018; Foster, 2019; Redero et al., 2020). These nine genes together occupy 5.85% (55,476 bp) of the entire SVXNc genome. Correspondingly, in the BNXNv genome, there are at least 14 genes encoding the proteins harboring the PKD and LamG domains, which occupy 6.15% (64,343 bp) of the entire BNXNv genome.

#### Giant membrane-anchored extracellular proteins

**Figure 5:**
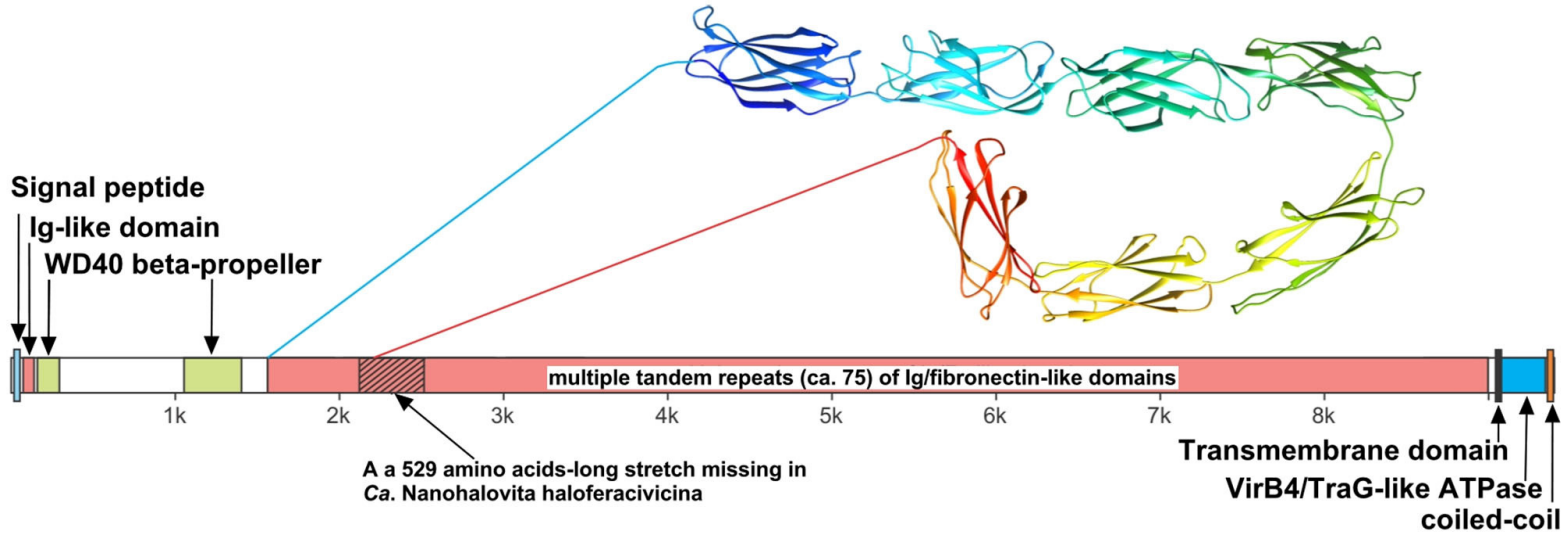
Domain structure of giant surface proteins BNXNv_0298 and SVXNc_0300. The giant protein of *Ca*. Nanohalovita BNXNv is 8,880 aa-long (gene is 26,643 bp-long) with a calculated molecular weight of 974,524 Da and an estimated pI of 4.11. The giant protein of *Ca*. Nanohalococcus SVXNc is 9,409 aa-long (gene is 28,230 bp-long) with a calculated molecular weight of 1,030,728 Da and an estimated pI of 4.03.

We believe that the giant surface proteins found in both genomes of the cultured nanohaloarchaea deserve special attention. Although these giant proteins were absent in the *Ca*. Nanohalobium constans genome (La Cono et al., 2020), their presence in many other nanohaloarchaea has been documented (see Hamm et al., 2019 for further references). The SVXNc_0300 cell-bound protein (9,409 amino acids) is the largest protein among all of the sequenced nanohaloarchaea and the largest protein ever found in any of the archaea that have been cultivated (Bolhuis et al., 2006). The BNXNv_0298 giant protein, also presumed to have a surface localization in the cellular envelope, is slightly shorter but is nevertheless enormous (8,880 amino acids). The predicted structures of these two giant proteins are somewhat similar each to other, but the BNXNv giant protein lacks 529 amino acids long stretch of tandem immunoglobulin (Ig-like)/fibronectin-like domains (indicated with a hatched box in Figure 5). Thus, for a detailed structural analysis, we have focused on the longest protein.

The N-terminus of the SVXNc_0300 protein apparently contains a Sec signal peptide, which would translocate the gigantic ectodomain to the outside of the cell. The signal peptide is followed by a single immunoglobulin-like (Ig-like) domain and then by two β-transducin (WD40-like) β-propeller domains, which contain pyrroloquinoline-quinine (PQQ)-like motifs. The WD40 repeat is a short structural motif of approximately 40 amino acids, often terminating in a tryptophan-aspartic acid (W-D) dipeptide. These repeats are found in extremely functionally diverse proteins and typically mediate interactions with various substrates, including proteins and DNA (Xu and Min, 2011). Nevertheless, the bulk of the SVXNc_0300 protein consists of tandem repetitions of Ig-like domains, and such tandem repeats are known (in invasins, intimins and S-layer proteins, and other proteins) to mediate adhesion (Nesta et al., 2012). We used AlphaFold2 to model a 700-amino acids region accommodating seven Ig-like domains (100 amino acids per one Ig-like domain), so there should be around 75 Ig-like domains in total. This extended region is followed by a transmembrane domain and then by the VirB4/TraG-like ATPase domain, which is one of the essential components of type IV secretion systems. The topology is consistent with the ATPase domain facing the cytosol (as would be expected). Finally, at the tip of the protein, there is a coiled-coil domain. Hamm et al. (2019) suggested that this domain is a restriction endonuclease, but this does not seem to be the case. In the homology searches, these authors obtained a match to a helicase domain of a type III restriction-methylation system and concluded that the corresponding domain of their SPEARE protein functions as a restriction enzyme. We reanalyzed the SPEARE protein of *Ca*. Nanohaloarchaeum antarcticus and found no endonuclease domain; instead, as in the cases of SVXNc_0300 and BNXNv_0298, the protein contains a well-conserved VirB4/TraG-like ATPase domain. Thus, the giant proteins in our nanohaloarchaea, along with the SPEARE proteins of *Ca*. Nanohaloarchaeum antarcticus and *Ca*. Halopetraeus SG9 (Oren and DiRuggiero, 2015), appear to be a part of the type IV secretion system, and further supports that it is a secretion system comes from the fact that the gene immediately downstream of the SVXNc_0300 giant protein gene (as well as BNXNv_0298) encodes another AAA+ ATPase (SVXNc_0299 and BNXNv_0297) of the VirB4/TraG- family. In addition to the AAA+ ATPase domain, the latter protein contains an N-terminal domain (PF03135.16), found in VirB4/TraG of *Rhizobium radiobacter* (P0A3W0), leaving little doubt that it is a secretion ATPase, rather than some other AAA+ ATPase. We then looked for other proteins encoded transcriptionally downstream of the SVXNc_0300 giant protein. Of special note is a proteasome activator protein SVXNc_0296, which could regulate the degradation of host proteins. Thus, we propose that the giant cell-bound proteins in nanohaloarchaea play a key role in subjugating the host cells by binding to their surface *via* the Ig-like domains and secreting yet unknown effectors into the host cells using the VirB4-like ATPase domain. We hypothesize that, in addition to the VirB4/TraG-like ATPase domain mentioned above, these giant proteins lack other enzymatic domains, in particular, the serine protease and restriction endonuclease domains, predicted in the previous study (Hamm et al., 2019). Accordingly, the naming of these giant proteins as SPEARE (after serine protease, adhesion, and restriction endonuclease) does not reflect their actual function. Rather than being the instrument responsible for intracellular invasion into the host (Hamm et al., 2023), these proteins likely function as secretion channels (straws) through which the nanohaloarchaeon can inject effector(s) that favorably affect the host metabolism. The latter possibility is supported by our recent results demonstrating a 50-fold increase in the production of glucose-6-phosphate in *Haloferax lucentense* cells associated with the SVXNc symbiont compared with axenic culture of the host (La Cono et al., 2023).

### Functional diversity emphasizing the uniqueness of the cultivated nanohaloarchaea

**Figure 6:**
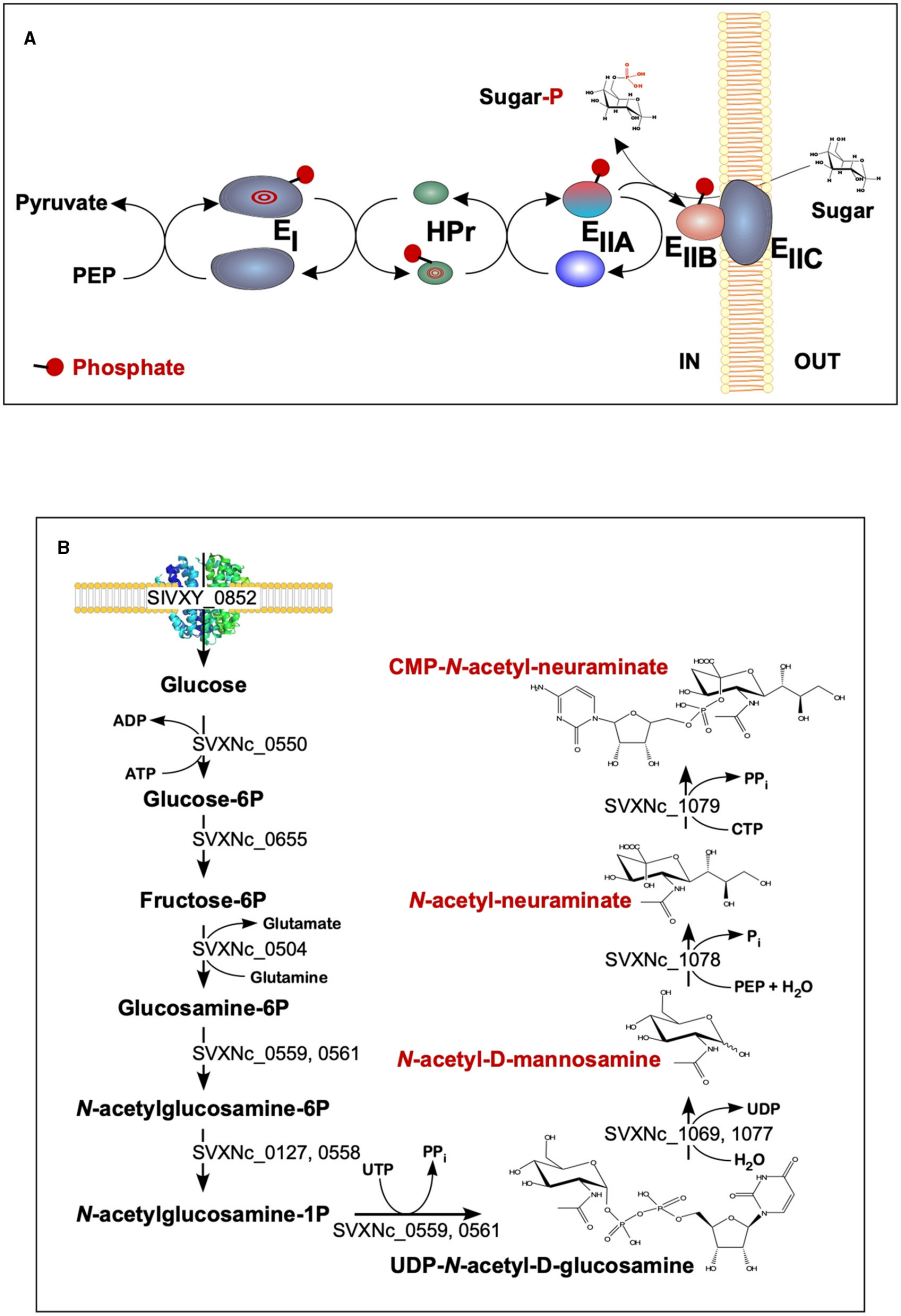
Schematic diagram of the BNXNv phosphotransferase (PTS) system **(A)** and the proposed pathway for sialic acid synthesis by SVXNc inferred from its genome analysis and transcriptome data **(B)**. The BNXNv PTS system seems to be responsible for the simultaneous transport and phosphorylation of sugar substrates. A series of enzyme intermediates, including EI (BNXNv_0770), HPr (BNXNv_0769), EIIA (BNXNv_0768), EIIB (BNXNv_0765), and EIIC (BNXNv_0767), were predicted to be phosphorylated.

The findings of the current study double the number (from 2 to 4) of the cultivated nanohaloarchaea and enrich the knowledge of their functional diversity. Although all four exhibited very similar fermentative-type catabolism, the novel cultivated nanohaloarchaea harbor unique genomic loci, neither observed before in the *Candidate* Nanohaloarchaeota phylum nor in any other members of the DPANN superphylum. Among them, noteworthy is the phosphoenolpyruvate phosphotransferase sugar transport system (PTS) in the *Ca*. Nanohalovita genome (BNXNv_0773-8). The PTS is present in many halophilic archaea, but it is not common for the entire DPANN superphylum. The only exception is the uncultivated nanohaloarchaeon M3_22 (Feng et al., 2021), which likely belongs to the same genus *Ca*. Nanohalovita (La Cono et al., 2023). In analogy to what is known as haloarchaeal PST (Eichler and Maupin-Furlow, 2013), the PTS of *Ca*. Nanohalovita was predicted to mediate the transfer of phosphoryl groups from phosphoenolpyruvate to imported sugars (Figure 6A). Although it was demonstrated that the PTS system in *Haloferax volcanii* is involved in the metabolism of fructose and/or galacticol (Eichler and Maupin-Furlow, 2013), participation of this system in transportation/metabolism of other monosaccharides in BNXNv cannot be ruled out.

Of particular interest is a complete set of genes responsible for the synthesis of sialic acid from glucose and UDP-*N*-acetylglucosamine found in the *SVXNc* genome (SVXNc_0559,0561,1069,1077-9) (Figure 6B). Apart from some mostly pathogenic bacteria, the ability to synthesize sialic acid has not been found in archaeal genomes, with the exception of the human intestinal methanogen *Methanobrevibacter smithii* and eight haloarchaeal species. The latter finding is very intriguing as it suggests that *Ca*. Nanohalococcus has developed mechanisms to disguise its surface with carbohydrate moieties that mimic those encountered in its haloarchaea host's glycan landscape, as was proposed elsewhere (Samuel et al., 2007). All these genes were found to be expressed in the SVXNc transcriptome (Supplementary Table S1), indicating that the synthesis of sialic acid occurs in this organism. Interestingly, the operon harboring the glycosyltransferase and *N*-acetylneuraminate synthases that are involved in the biosynthesis of sialic acid was found within the predicted horizontally transferred second genomic island (Figure 3).

The ability to adapt to environmental changes, not least in relation to stress responses and interactions with host organisms, requires ectosymbiotic nanohaloarchaea to control the expression of various host genes. It appears that in the newly characterized nanohaloarchaea, this occurs *via* at least two different mechanisms that modulate certain cellular pathways.*Ca*. Nanohalococcus has an actively expressed *cyaB* gene (SVXNc_0489, value of 344 ± 76 FPKM [fragments per kilobase of gene per million mapped fragments]) encoding an adenylate cyclase class IV, which synthesizes the intracellular signal transmitter cAMP. The role of cyclic nucleotides as second messengers for intracellular signal transduction is well described in bacteria and archaea, including haloarchaea (see Braun et al., 2019 for further references), where it was shown that cAMP serves as a catabolite de-repression signal that triggers the expression of many metabolic pathways associated with the cell cycle and osmotic adjustment (Baumann et al., 2007). To our knowledge, the presence of adenylate cyclase class IV has not been reported before in any of the genomes of DPANN members.

Analyzing both genomic and transcriptomic data, at least five genes of non-coding regulatory RNA (ncRNA) were predicted in the SVXNc genome by two-dimensional structures typical of ncRNA molecules (Supplementary Table S3). They exhibited an extremely high level of expression that was two to three orders of magnitude higher than the average level calculated for all other genes. Homologous versions of the genes SVXNc_nc0001 and SVXNc_nc0002 with characteristic 2D structures were also found in the *Ca*. Nanohalovita genome. However, the search for similar ncRNAs using BLAST did not give significant matches with any of the prokaryotes that have been sequenced, clearly indicating their organism-specific nature. Non-coding regulatory RNAs play an important role in the regulation of gene transcription and mRNA translation in archaea (Gelsinger and DiRuggiero, 2018). Using the StructRNAfinder web server (Arias-Carrasco et al., 2018), only SVXNc_nc0002 was predicted with high confidence (E value of 2e^−32^) to be an archaeal signal recognition particle RNA (SRP). The function of SRP is to delay protein translation until the ribosome-bound SRP has an opportunity to associate with the membrane-resident SRP receptor, such as the Sec-translocating system. The other four ncRNAs were not affiliated with any known families of ncRNAs, although their 2D structure resembled the structures of ribosome-associated ncRNA (rancRNA) molecules. All of them have typical rancRNA H/ACA regions, consisting of two hairpins and two single-stranded regions termed a hairpin–hinge–hairpin–tail structure (Supplementary Table S3).

## Conclusion

This study has doubled the number of cultivated genera representing the phylum *Ca*. Nanohaloarchaeota, an extremely halophilic archaeal lineage within the DPANN superphylum. Omics analyses of new nanohaloarchaea showed that, despite their rudimentary catabolic capabilities and the absence of a minimal set of enzymes required for the biosynthesis of nucleotides, amino acids, lipids, vitamins, and cofactors, they possess numerous unique adaptive mechanisms, necessary for their ectosymbiotic lifestyle. All genomic loci for the latter have likely been acquired through the horizontal gene transfer from other members of an extremely halophilic microbial community, suggesting a high evolutionary dynamism and plasticity of their reduced genomes. In addition, all our cultured nanohaloarchaea illustrate their common evolutionary history of adaptation to the hypersaline environment and interspecies interactions with polysaccharidolytic haloarchaea, which can now be confidentially assigned to the classical types of either mutualistic symbiosis or pure commensalism.

## Experimental procedures

### Laboratory cultivation of archaeal strains

Both nanohaloarchaeal strains, *Ca*. Nanohalococcus occultus SVXNc and *Ca*. Nanohalovita haloferacivicina BNXNv, were cultivated in binary cultures on the LC medium (La Cono et al., 2020), supplemented with D-xylose (2 g L^−1^) at 37°C without shaking together with their corresponding hosts, *Haloferax lucentense* SVX82 and BNX82, respectively (La Cono et al., 2023). The presence of nanohaloarchaea in the culture was monitored by light microscopy and PCR amplification with the nanohaloarchaea-specific primers as described previously (La Cono et al., 2020, 2023).

### Field emission scanning electron microscopy

The grown cells were fixed with 2% (v/v, final concentration) freshly prepared paraformaldehyde. The fixative was removed by washing twice with LC mineral medium. Following that, a final fixation with aqueous osmium tetroxide was carried out (four parts LC mineral medium and one part 5% [w/v] aqueous osmium tetroxide) for 30 min at 25°C. The fixed material was then washed with LC mineral medium and placed onto poly-L-lysine-coated coverslips for 10 min, followed by treatment with 1% (v/v) glutaraldehyde to cross-link microbes with poly-L-lysine coating. This step prevents cells from being washed away during the dehydration and critical-point drying of the attached microorganisms. Dehydrating was achieved using a series of acetone–water mixtures and pure acetone (10, 30, 50, 70, 90, and 100% [v/v] acetone) on ice for 10 min for each step. Once in the 100% acetone, samples were allowed to reach 25°C (evaporation of acetone has cooled the sample), replenishing with fresh 100% acetone. Samples were then subjected to critical-point drying with liquid CO_2_ (CPD 030, Bal-Tec, Liechtenstein). Dried samples were covered with a gold–palladium film by sputter coating (SCD 500 Bal-Tec, Liechtenstein) before examination in a field-emission scanning electron microscope Zeiss Merlin (Carl Zeiss, Oberkochen) using the Everhart-Thornley SE detector and the in-lens secondary electron detector in a 50:50 ratio with an acceleration voltage of 5 kV. Contrast and brightness were adjusted with Adobe Photoshop CS5.

### DNA extraction and sequencing

Genome sequencing assembly and curation as well as the phylogenetic, phylogenomic, and proteomic analyses are described in detail in our precedent study (La Cono et al., 2020, 2023). Shortly, the DNA for genome sequencing was extracted from 2.0 ml of corresponding co-cultures collected at a fixed time (120 h), corresponding to mid-log phases of growth using a GNOME DNA kit (MP Biomedicals, USA). Extracted DNA was dissolved in 50 μl of TE buffer (10 mM Tris-HCl, 1 mM EDTA [pH 7.5]) and quantified using a NanoDrop ND-1000 spectrophotometer (Celbio). The quality of the extracted DNA was checked by electrophoresis in a 1.0% agarose gel. Whole-genome shotgun sequencing of the trinary, binary, and axenic cultures was done by FISABIO (Valencia, Spain) using the Illumina^®^ MiSeq System platform (San Diego, CA, United States) with 2 × 300 bp short insert paired-end libraries (MiSeq Reagent Kit v3). FISABIO also performed the quality assessment and the sequence joining (forward R1 and reverse R2). Quality assessment was performed with the PRINSEQ-lite program using the following parameters: Min_length:50 bp trim_qual_right:30 bp trim_qual_typ:mean, trim_qual_window:20 bp. Forward and reverse reads were joined by the FLASH program applying default parameters. All two genomes were assembled as single circular chromosomes and analyzed as described previously (La Cono et al., 2020; Sorokin et al., 2021).

### Total RNA extraction and sequencing

For total RNA extraction, 70 ml of the binary culture of *Ca*. Nanohalococcus occultus SVXNc + Haloferax lucertense SVX82 (three replicates) was cultivated, using the LC medium containing D-xylose (2 g L^−1^), statically at 37°C for 120 h, corresponding to the mid-log phase of growth. After centrifugation at 10,000 × *g* for 120 min, the biomass from 5 ml of grown culture was collected and total RNA was extracted using MasterPure Complete DNA and RNA purification RNA kits (Epicenter). Total RNA was stored in isopropanol at −20°C before precipitation. RNA was resuspended in 50 μl of RNase-free water. Extracted RNA was treated with a TURBO DNA-free kit (Ambion) to eliminate any residual DNA from the final elution. The quality and concentration of RNA samples were determined using Qubit 3.0 fluorometer (Thermo Fisher Scientific, Italy). The metatranscriptome analysis was performed by FISABIO (Valencia, Spain) using the Illumina^®^ NextSeq Mid Output platform (San Diego, CA, United States) with 2 × 100 bp short insert paired-end libraries (NextSeq Reagent Kit v2.5). FISABIO also performed the quality assessment and the sequence joining (forward R1 and reverse R2). Quality assessment was performed with the PRINSEQ-lite program using the following parameters: min_length: 50 bp; trim_qual_right: 30 bp; trim_qual_type: mean; and trim_qual_window: 20 bp.

### Whole-genome sequence analysis and annotation

Metabolic pathways were predicted based on the annotated whole-genome sequences of the strains using the PathoLogic tool of the program Pathway Tools 26.0 (Paley et al., 2021). Information about enzyme encoding genes and the related metabolic pathways was extracted using the SmartTables function of the program. The network of metabolic pathways was visualized using Cytoscape 3.9.1 (https://cytoscape.org/). Horizontally acquired genomic islands (GIs) were identified using SeqWord Genomic Island Sniffer (Bezuidt et al., 2009). Genomic comparisons and synteny were visualized using *Circos* software (Krzywinski et al., 2009) and Blast Dot Plot graph obtained by the RAST server (Aziz et al., 2008; Overbeek et al., 2014; Brettin et al., 2015). Percentages of amino acid identity levels used as the input for Circos visualization and the Blast Dot Plot graph were obtained by the RAST server. Average nucleotide identity (ANI) was calculated for each genome couple compared using the ANI calculator (http://enve-omics.ce.gatech.edu/ani/index) as described previously (Goris et al., 2007; Rodriguez and Konstantinidis, 2014). The CRISPRfinder online program (https://crispr.i2bc.paris-saclay.fr/cgi-bin/crispr/advRunCRISPRFinder.cgi) was used to detect CRISPRs system in all sequenced and annotated genomes of nanohaloarchaea. The NetNGlyc-1.0 online program (https://services.healthtech.dtu.dk/services/NetNGlyc-1.0) was used to predict N-glycosylation sites in nanohaloarchaeal proteins using artificial neural networks that examine the sequence context of Asn-Xaa-Ser/Thr sequons (Gupta and Brunak, 2002). The whole-genome reference sequence of the strain *Ca*. Nanohalococcus occultus SVXNc [CP107255] was indexed using the *buildindex* function of the Rsubreads package (Bioconducter, www.bioconductor.org). The obtained Illumina RNA reads were mapped to the reference sequence by the function *align* of the Rsubread package. The numbers of reads aligned against each coding sequence of the reference genomes were counted by the function *featureCounts* of the Rsubread package and then normalized by the lengths of the coding sequences to Reads per Kilobase per Million reads (RPKM) values shown in Supplementary Table S1. The prediction of ncRNA was confirmed by modeling the 2D structures of the ncRNA molecules using the RNAfold WebServer (http://rna.tbi.univie.ac.at/cgi-bin/RNAWebSuite/RNAfold.cgi).

### Genome methylation profiling

For the prediction of genome methylation profiles, the PacBio reads were mapped against the whole-genome sequence of the strain *Ca*. Nanohalococcus occultus SVXNc using pbmm2 aligner of the software package SMRT Link v10.1.0.119588 (https://www.pacb.com/support/software-downloads/) followed by the prediction of methylated nucleotides and methylation motifs using ipdSummary and motifMaker tools of the SMRT Link package. The following command lines were used to run the pipelines on the server:

pbmm2 index *path_to_reference_FASTA_file path_to_reference _index_MMI_file*.
pbmm2 align –sort *path_to_reference_index_MMI_file path_to_read_database_XML_file path_to_output_BAM_file*.
ipdSummary *path_to_output_BAM_file* –reference *path_to_reference_FASTA_file* –identify m6A, m4C –gff *path_to_output_GFF_file*.
motifMaker find –fasta *path_to_reference_FASTA_file* –gff *path_to_output_GFF_file* –minScore 30 –output *path_to_motif_output_CSV_file*.

## Data availability statement

The datasets presented in this study can be found in online repositories. The names of the repository/repositories and accession number(s) can be found in the article/Supplementary material.

## Author contributions

MY, FC, VL, ES, and LM were responsible for microbiology work. OR, EM, and MY designed the research. OR, EM, FS, MK, GL, and MY carried out bioinformatics analyses and data interpretation. MR performed FESEM analysis. OR, EM, FS, VL, FC, and GL were responsible for genome sequencing, assembling, and curation. MY, OR, and MK wrote the manuscript, with the contribution of all authors. All authors contributed to the article and approved the submitted version.
