# Supplementary material for: Functional diversity of nanohaloarchaea within xylan-degrading consortia
Source: Front Microbiol. 2023 May 31;14:1182464. doi: 10.3389/fmicb.2023.1182464 (PMC10266531; doi:10.3389/fmicb.2023.1182464)
Supplement: Supplementary file 2 [file Data_Sheet_1.PDF]

Supplementary Table S2: Location of methylated GD*GC* HC motifs in genome of *Ca.* N. occultus SVXNc.

Motif	Strand	Location	Type	First repeat		Second repeat		Annotation
				Coverage	Qv score	Coverage	Qv score	
GAGCAC	DIR	1630..1635	m4C	74	57	215	198	SIVXY_0002 [1352..2963]; HYS2; Archaeal DNA polymerase II, small subunit/DNA polymerase delta, subunit B
GGGCTC	DIR	10723..10728	m4C	67	51	265	174	non-coding
GAGCTC	DIR	13030..13035	m4C	104	40	337	193	SIVXY_0016 [12962..13766]; hypothetical protein
GTGCCC	DIR	21303..21308	m4C	56	46	195	226	non-coding
GAGCCC	DIR	33818..33823	m4C	71	56	157	162	non-coding
GAGCTC	DIR	67806..67811	m4C	105	75	235	184	non-coding
GAGCTC	DIR	73120..73125	m4C	118	81	298	215	SIVXY_0082 [73097..73265]; hypothetical protein
GAGCAC	DIR	78832..78837	m4C	103	64	208	190	SIVXY_0090 [78552..79161]; hypothetical protein
GTGCTC	DIR	88475..88480	m4C	107	64	276	217	non-coding
GTGCTC	DIR	88550..88555	m4C	91	65	320	214	non-coding
GTGCTC	DIR	89223..89228	m4C	69	60	220	213	non-coding
GAGCTC	DIR	89777..89782	m4C	94	48	270	202	non-coding
GGGCTC	DIR	133150..133155	m4C	115	69	235	168	non-coding
GAGCTC	DIR	151394..151399	m4C	118	77	262	197	non-coding
GAGCTC	DIR	151666..151671	m4C	110	74	227	185	non-coding
GTGCTC	DIR	162076..162081	m4C	65	51	230	240	non-coding
GTGCCC	DIR	162418..162423	m4C	35	44	120	173	non-coding
GAGCCC	DIR	184524..184529	m4C	69	56	159	163	non-coding
GAGCAC	DIR	184533..184538	m4C	67	62	164	195	non-coding
GAGCTC	DIR	224035..224040	m4C	91	70	336	209	SIVXY_0252 [223887..226734]; hypothetical protein
GTGCTC	DIR	248361..248366	m4C	106	64	311	216	non-coding
GTGCTC	DIR	248546..248551	m4C	124	69	253	221	non-coding
GGGCTC	DIR	253198..253203	m4C	79	48	310	197	non-coding
GAGCTC	DIR	391086..391091	m4C	126	75	242	179	Upstream SIVXY_0413 [390545..391103]; TrmJ; tRNA C32,U32 (ribose-2'-O)-methylase TrmJ or a related methyltransferase
GAGCTC	DIR	421928..421933	m4C	69	47	282	196	non-coding
GGGCTC	DIR	425662..425667	m4C	65	42	261	170	non-coding
GGGCTC	DIR	426710..426715	m4C	70	44	199	152	non-coding
GAGCTC	DIR	432440..432445	m4C	109	64	207	120	non-coding
GAGCTC	DIR	446577..446582	m4C	108	70	252	179	non-coding
GGGCTC	DIR	453119..453124	m4C	122	63	246	166	SIVXY_0492 [452742..453414]; Alpha/beta superfamily hydrolase
GAGCAC	DIR	454683..454688	m4C	61	60	215	184	SIVXY_0494 [454662..454938]; hypothetical protein
GAGCTC	DIR	456052..456057	m4C	83	71	282	187	non-coding
GGGCCC	DIR	510635..510640	m4C	61	43	193	193	SIVXY_0566 [510545..511538]; DPH2; Diphthamide synthase subunit DPH2
GAGCTC	DIR	513199..513204	m4C	85	55	343	212	non-coding
GGGCTC	DIR	515870..515875	m4C	112	78	276	204	non-coding
GGGCCC	DIR	549253..549258	m4C	34	39	125	133	non-coding
GGGCTC	DIR	549413..549418	m4C	84	45	254	144	non-coding
GGGCTC	DIR	558619..558624	m4C	96	53	166	126	SIVXY_0632 [558586..559669]; FtsZ; Cell division GTPase
GTGCCC	DIR	575442..575447	m4C	91	56	170	162	non-coding
GAGCTC	DIR	576094..576099	m4C	70	59	131	180	non-coding
GAGCAC	DIR	611287..611292	m4C	106	81	220	159	SIVXY_0690 [610929..611316]; NikR; Transcriptional regulator, CopG/Arc/MetJ family (DNA-binding and a metal-binding domains)
GAGCTC	DIR	627802..627807	m4C	149	77	184	127	SIVXY_0710 [627251..627815]; SPT15; TATA-box binding protein (TBP), component of TFIID and TFIIIB
GAGCTC	DIR	632616..632621	m4C	105	65	257	150	SIVXY_0717 [632449..632644]; hypothetical protein
GGGCCC	DIR	638618..638623	m4C	98	59	163	142	non-coding
GAGCAC	DIR	638640..638645	m4C	86	63	210	159	non-coding
GGGCCC	DIR	638745..638750	m4C	81	59	157	144	non-coding
GGGCTC	DIR	648229..648234	m4C	124	73	359	207	non-coding
GTGCTC	DIR	651728..651733	m4C	115	67	223	180	non-coding
GAGCCC	DIR	678786..678791	m4C	74	62	162	147	non-coding
GAGCTC	DIR	716562..716567	m4C	125	66	305	220	non-coding
GAGCTC	DIR	752396..752401	m4C	117	69	266	169	non-coding
GAGCAC	DIR	797917..797922	m4C	68	70	222	183	SIVXY_0907 [797661..798258]; phoE; Broad specificity phosphatase PhoE or related phosphatase
GAGCTC	DIR	805146..805151	m4C	94	62	250	177	SIVXY_0920 [804425..806963]; Smc; Chromosome segregation ATPase
GGGCCC	DIR	832178..832183	m4C	81	47	165	183	non-coding
GGGCAC	DIR	833679..833684	m4C	78	50	178	256	non-coding
GGGCAC	DIR	833852..833857	m4C	80	51	214	284	non-coding
GGGCTC	DIR	833870..833875	m4C	87	51	251	269	non-coding
GAGCTC	DIR	835140..835145	m4C	75	55	246	279	non-coding
GGGCTC	DIR	857158..857163	m4C	124	79	305	215	non-coding
GAGCCC	DIR	871467..871472	m4C	64	79	127	169	non-coding
GGGCCC	DIR	885021..885026	m4C	51	57	141	169	non-coding
GAGCTC	DIR	886340..886345	m4C	99	67	255	175	non-coding
GAGCTC	DIR	911901..911906	m4C	117	70	285	198	non-coding
GAGCTC	DIR	912386..912391	m4C	122	68	303	197	non-coding
GTGCTC	DIR	935762..935767	m4C	125	70	296	199	non-coding
GTGCTC	DIR	936388..936393	m4C	87	57	253	202	non-coding
GAGCTC	DIR	938869..938874	m4C	91	61	260	204	non-coding
GAGCCC	DIR	938890..938895	m4C	54	55	98	181	non-coding
GGGCTC	DIR	939421..939426	m4C	83	57	275	193	non-coding
GAGCCC	REV	939421..939426	m4C	55	54	180	197	SIVXY_1082 [938158..939481]; BetC; Choline-sulfatase; Arylsulfatase A or related enzyme
GGGCTC	REV	938890..938895	m4C	77	54	315	195	SIVXY_1082 [938158..939481]; BetC; Choline-sulfatase; Arylsulfatase A or related enzyme
GAGCTC	REV	938869..938874	m4C	79	55	319	201	SIVXY_1082 [938158..939481]; BetC; Choline-sulfatase; Arylsulfatase A or related enzyme
GAGCAC	REV	936388..936393	m4C	74	57	230	219	SIVXY_1080 [935850..937239]; hypothetical protein
GAGCAC	REV	935762..935767	m4C	60	68	234	223	SIVXY_1079 [935167..935854]; NeuA; CMP-N-acetylneuraminic acid synthetase

GAGCTC	REV	912386..912391	m4C	77	67	239	186	SIVXY_1056 [911711..912566]; Endonuclease YncB, thermonuclease family
GAGCTC	REV	911901..911906	m4C	103	60	245	186	SIVXY_1056 [911711..912566]; Endonuclease YncB, thermonuclease family
GAGCTC	REV	886340..886345	m4C	139	73	254	173	SIVXY_1024 [886106..886868]; hypothetical protein
GGGCCC	REV	885021..885026	m4C	68	63	150	187	Upstream SIVXY_1022 [884722..884965]; hypothetical protein
GGGCTC	REV	871467..871472	m4C	118	89	241	187	SIVXY_1003 [871395..871653]; hypothetical protein
GAGCCC	REV	857158..857163	m4C	99	82	230	214	non-coding
GAGCTC	REV	835140..835145	m4C	119	61	244	287	non-coding
GAGCCC	REV	833870..833875	m4C	94	57	135	286	non-coding
GTGCCC	REV	833852..833857	m4C	79	56	93	289	non-coding
GTGCCC	REV	833679..833684	m4C	54	54	107	279	non-coding
GGGCCC	REV	832178..832183	m4C	72	52	172	190	non-coding
GAGCTC	REV	805146..805151	m4C	106	61	250	182	non-coding
GTGCTC	REV	797917..797922	m4C	102	69	280	177	non-coding
GAGCTC	REV	752396..752401	m4C	101	69	257	166	SIVXY_0842 [752172..753321]; Serine protease
GAGCTC	REV	716562..716567	m4C	148	69	303	210	SIVXY_0807 [714948..719196]; LamG domain-containing protein
GGGCTC	REV	678786..678791	m4C	116	71	206	157	SIVXY_0785 [677066..679901]; UvrA; Excinuclease ABC subunit A, ATPase
GAGCAC	REV	651728..651733	m4C	94	61	174	197	SIVXY_0752 [650708..651740]; DNA modification methylase, adenine-specific
GAGCCC	REV	648229..648234	m4C	97	78	255	223	non-coding
GGGCCC	REV	638745..638750	m4C	76	60	161	162	non-coding
GTGCTC	REV	638640..638645	m4C	100	58	222	160	non-coding
GGGCCC	REV	638618..638623	m4C	81	61	202	163	non-coding
GAGCTC	REV	632616..632621	m4C	136	66	215	151	non-coding
GAGCTC	REV	627802..627807	m4C	117	72	166	122	non-coding
GTGCTC	REV	611287..611292	m4C	132	78	189	147	non-coding
GAGCTC	REV	576094..576099	m4C	102	58	242	171	SIVXY_0650 [575340..576792]; PpsA; Phosphoenolpyruvate synthase/pyruvate phosphate dikinase
GGGCAC	REV	575442..575447	m4C	66	63	145	177	SIVXY_0650 [575340..576792]; PpsA; Phosphoenolpyruvate synthase/pyruvate phosphate dikinase
GAGCCC	REV	558619..558624	m4C	69	55	145	122	non-coding
GAGCCC	REV	549413..549418	m4C	36	40	157	143	non-coding
GGGCCC	REV	549253..549258	m4C	37	37	173	141	non-coding
GAGCCC	REV	515870..515875	m4C	66	80	214	215	non-coding
GAGCTC	REV	513199..513204	m4C	84	56	330	223	SIVXY_0568 [512800..513445]; hypothetical protein
GGGCCC	REV	510635..510640	m4C	47	48	234	217	non-coding
GAGCTC	REV	456052..456057	m4C	128	71	282	185	SIVXY_0497 [455672..456110]; hypothetical protein
GTGCTC	REV	454683..454688	m4C	119	64	252	178	non-coding
GAGCCC	REV	453119..453124	m4C	73	59	160	166	non-coding
GAGCTC	REV	446577..446582	m4C	97	67	252	180	SIVXY_0485 [446274..447108]; HflC; Membrane protease subunit, stomatin/prohibitin-like; Membrane protease subunit, stomatin/prohibitin homolog
GAGCTC	REV	432440..432445	m4C	97	57	139	127	SIVXY_0470 [432277..432478]; hypothetical protein
GAGCCC	REV	426710..426715	m4C	67	47	129	151	Upstream SIVXY_0457 [426438..426678]; hypothetical protein
GAGCCC	REV	425662..425667	m4C	59	43	143	197	non-coding
GAGCTC	REV	421928..421933	m4C	93	50	348	194	SIVXY_0449 [421876..422872]; Predicted aminoglycoside phosphotransferase
GAGCTC	REV	391086..391091	m4C	116	74	240	187	non-coding
GAGCCC	REV	253198..253203	m4C	61	46	267	209	non-coding
GAGCAC	REV	248546..248551	m4C	78	67	189	225	SIVXY_0272 [245923..248992]; HerA helicase
GAGCAC	REV	248361..248366	m4C	96	65	242	221	SIVXY_0272 [245923..248992]; HerA helicase
GAGCTC	REV	224035..224040	m4C	116	71	302	204	non-coding
GTGCTC	REV	184533..184538	m4C	94	60	228	204	non-coding
GGGCTC	REV	184524..184529	m4C	101	62	277	197	non-coding
GGGCAC	REV	162418..162423	m4C	76	54	199	208	SIVXY_0187 [161190..162459]; TEF1; Translation elongation factor EF-1 alpha, GTPase
GAGCAC	REV	162076..162081	m4C	79	57	184	259	SIVXY_0187 [161190..162459]; TEF1; Translation elongation factor EF-1 alpha, GTPase
GAGCTC	REV	151666..151671	m4C	106	73	245	196	SIVXY_0170 [151388..151907]; hypothetical protein
GAGCTC	REV	151394..151399	m4C	107	80	283	201	Upstream SIVXY_0169 [150843..151392]; RdgB; Inosine/xanthosine triphosphate pyrophosphatase,all-alpha NTP-PPase family
GAGCCC	REV	133150..133155	m4C	135	81	174	172	SIVXY_0150 [131265..134931]; BRR2; Replicative superfamily II helicase
GAGCTC	REV	89777..89782	m4C	90	52	241	194	SIVXY_0101 [87570..90399]; hypothetical protein
GAGCAC	REV	89223..89228	m4C	75	58	204	209	SIVXY_0101 [87570..90399]; hypothetical protein
GAGCAC	REV	88550..88555	m4C	80	67	211	212	SIVXY_0101 [87570..90399]; hypothetical protein
GAGCAC	REV	88475..88480	m4C	79	69	187	217	SIVXY_0101 [87570..90399]; hypothetical protein
GTGCTC	REV	78832..78837	m4C	92	63	211	192	non-coding
GAGCTC	REV	73120..73125	m4C	122	86	297	220	non-coding
GAGCTC	REV	67806..67811	m4C	139	77	247	195	Upstream SIVXY_0073 [65509..67780]; Cdc48; ATPase of the AAA class , CDC48 family; ATPase of the AAA+ class , CDC48 family
GGGCTC	REV	33818..33823	m4C	77	61	247	182	SIVXY_0040 [33354..33981]; Uncharacterized membrane protein
GGGCAC	REV	21303..21308	m4C	63	53	234	229	SIVXY_0027 [21158..22109]; RecA; RecA/RadA recombinase
GAGCTC	REV	13030..13035	m4C	76	41	223	199	non-coding
GAGCCC	REV	10723..10728	m4C	84	55	172	183	non-coding
GTGCTC	REV	1630..1635	m4C	100	54	270	199	non-coding
