# Supplementary material for: Functional diversity of nanohaloarchaea within xylan-degrading consortia
Source: Front Microbiol. 2023 May 31;14:1182464. doi: 10.3389/fmicb.2023.1182464 (PMC10266531; doi:10.3389/fmicb.2023.1182464)
Supplement: Supplementary file 3 [file Data_Sheet_2.docx]

“*Human subtlety will never devise an invention more beautiful, more simple or more direct than does Nature, because in her inventions, nothing is lacking and nothing is superfluous*.”

**Leonardo da Vinci (1452–1519)**

**SUPPLEMENTARY INFORMATION FOR**

**Functional diversity of nanohaloarchaea within xylan-degrading consortia: a view from the genome**

**Oleg Reva^1†^, Enzo Messina^2†^, Violetta La Cono^3^, Francesca Crisafi^3^, Francesco Smedile^3^, Gina La Spada^3^, Laura Marturano^3^, Elena A. Selivanova^4^, Manfred Rohde^5^, Mart Krupovic^6^, John E. Hallsworth^7^, Michail M. Yakimov^3^***

Corresponding author. Email: [mikhail.iakimov@cnr.it](mailto:mikhail.iakimov@cnr.it)

**This PDF file includes:**

**Supplementary Tables 1 and 2 are provided as separated files**

**Supplementary Table S3 2**

**Supplementary Figure S1 4**

**Legend for SI Extended Datasets S1 * 7**

**Table S3.** Non-coding regulatory ncRNAs found in *Ca*. Nanohalococcus occultus SVXNc genome and confirmed by transcriptome analysis.

| **Gene** | **Length, bp** | **Normalized gene expression** | **ncRNA family,**  **E Value** | **Predicted 2D structure** |
| --- | --- | --- | --- | --- |
| **SVXNc_nc0001** | **322** | **328.46** | **Unknown family of putative ribosome associated ncRNA (rancRNA) with typical H/ACA boxes** |  |
| **SVXNc_nc0002** | **301** | **1343.73** | **Archaea_SRP (RF01857): archaeal signal recognition particle RNA, 2e-32** |  |
| **SVXNc_nc0003** | **115** | **101.06** | **Unknown family of putative ribosome associated ncRNA (rancRNA) with typical H/ACA boxes** |  |
| **SVXNc_nc0004** | **140** | **283.76** | **Unknown family of putative ribosome associated ncRNA (rancRNA) with typical H/ACA boxes** |  |
| **SVXNc_nc0005** | **155** | **11.93** | **Unknown family of H/ACA rancRNA** |  |

**Sequences:**

**SVXNc_nc0001**

CUUUUUGUGUUUGUACGUGGUGUUGAGCGUCUUGAAAUUUUCCAGCGCCGGCCCAUACCUUGCAAGCAUGAACCGGCGUUAAUUCGGAGCCUGUAUCGAUGCAAGGACGACAGGUUAAGCUCCGAGUCCAGUUUUGUUGAGUUUCGCGGUCGGCGCGUUGGUCCAAGUCAGGACCAAGGCUUCAUCCCACUCUACUCUAGGAUAAUAUUGUAGAAAGCCUUGGCCUUGUGGUUAUAGGUAAAUCUCCCCAGAUCCACGGACAUCCAAACGUUUCUAGGGAAUGCGGUUUGAGUAACCAAGGCCAUGUUUUCAGGUCAAUAUU

**SVXNc_nc0002** UUAACGUAGAUGGGGAAAAAUAGUGGUUCCCGGAGGUGGACGCCGGUUCAACGCCGAGAACUCCCGAGACCUGUCUCAGAUCCGCCACCCUCCGAACAUGCCGAGGACUGCUUAGGGCUGCUCCCGUCAAGGCCUGACCCGGUUCACAGCCCCCAACAUCCCGGAGGACGGAUCGUCAACAACAGGUUUCAGGGCUCGAACGCCGAACGACAGCGUCGCCCUCUGGUUGACGGCUCCGGCAUUAACUGAGGAUUUCCGGAAAGAGGCGACCCCGAGCCGCCCGGCCUACCAAGUUAGUAUC

**SVXNc_nc0003** UUUUUGAGCGGUUUCCUAAGCCGCUUUUGAGACAUCACCUCGUCGAACAAAUCAAGGUUUUUGGCGUUUCAAUCUGUUUUAGUGUUUUUUCGUUUUGUUUGUUGGCGAUUGUGUG

**SVXNc_nc0004**

AUGUUUGCUCCGAAUUUUUUCGGCCGAGGUCUGCCCACCGGAGCCUCCUGAGUUGAAAGCUCCGUCAGAUCGUCUCAGCCUACUUAUCCUGUACUACAUAUACAGGAUGUUCGGAAUAUUAGAUUGAAUAGUGUUUUUGC

**SVXNc_nc0005**

UUUUGAUUCCCACGCUUUCUAUUCAUCUUGGCUGGUUAUUAAUUCUAGGCGAAUACACAGGAGUUGUGGCAGUAUCCCUCGGUGGCUUGGACGUAGUCUUCGGACGUGUUAACCCUUUUUCCACAGACUACACAUAGGCCGAGCUUGACGACGGU

**Figure S1.** Overview of metabolic potential of the two sequenced symbiotic nanoarchaea *Ca*. Nanohalococcus occultus SVXNc and *Ca*. Nanohalovita haloferacivicina BNXNv. Numbered nodes correspond to intermediate compounds of metabolic pathways. A list of full names of the compounds is given below. Metabolic reactions shared by the microorganisms are depicted by blue graphs. Metabolic reactions specific for the strain SVXNc are shown as pink graphs and those specific for BNXNv are shown by orange graphs. The names of genes catalyzing the respective reactions are shown in the same color and mentioned in SI text

**List of compounds in Figure #SX**

1 large-branched glucan

2 maltose

3 n-maltodextrin

4 glycogen

5 glucose

6 D-glucose 6-phosphate

7 fructose 6-phosphate

8 fructose 1,6-bisphosphate

9 D-glyceraldehyde 3-phosphate

10 glycerone phosphate

11 3-phospho-D-glyceroyl phosphate

12 3-phospho-D-glycerate

13 2-phospho-D-glycerate

14 phosphoenolpyruvate

15 pyruvate

16 acetyl-CoA

17 (3S)-citryl-CoA

18 oxaloacetate

19 malate

20 citryl-holo [citrate lyase acyl-carrier protein]

21 acetyl-holo [citrate lyase acyl-carrier protein]

22 aliphatic N-acetyl-diamine

23 aliphatic a,o-diamine

24 S-acetyldihydrolipoamide

25 dihydrolipoamide

26 (R)-lactate

27 propanoyl-CoA

28 propanoate

29 acetate

30 N-acetyl-L-ornithine

31 L-ornithine

32 N-acetyl-L-citrulline

33 L-citrulline

34 2-oxoglutarate

35 succinyl-CoA

36 succinate

37 N2-succinylglutamate

38 L-glutamate

39 3-sulfopyruvate

40 L-cysteate

41 L-aspartate

42 L-tyrosine

43 aromatic 2-oxo-acid

44 L-phenylalanine

45 3-(4-hydroxyphenyl)pyruvate

46 3-phenyl-2-oxopropanoate

47 prephenate

48 chorismate

49 (indol-3-yl)pyruvate

50 L-tryptophan

51 D-alanine

52 5,7-diacetamido-3,5,7,9-tetradeoxy-D-glycero-D-galacto-nonulopyranosonate

53 2,4-diacetamido-2,4,6-trideoxy-a-D-mannopyranose

54 CMP-N,N'-diacetyllegionaminate

55 N-acetyl-b-neuraminate

56 CMP-N-acetyl-b-neuraminate

57 N-acetyl-D-mannosamine

58 UDP-N-acetyl-D-glucosamine

59 N-acetyl-a-D-glucosaminyl-diphosphodolichol

60 N-acetyl-a-D-glucosamine 1-phosphate

61 N-acetyl-a-D-glucosamine 6-phosphate

62 b-D-GlcNAc-(1->4)-Mur2Ac(oyl-L-Ala-g-D-Glu-DAP-D-Ala-D-Ala)-PP-Und

63 Und-PP-Mur2Ac-L-Ala-g-D-Glu-DAP-D-Ala-D-Ala

64 D-sedoheptulose 1-phosphate

65 D-erythrose

66 b-D-fructofuranose 1-phosphate

67 D-glyceraldehyde

68 D-sedoheptulose 1,7-bisphosphate

69 D-erythrose 4-phosphate

70 4-phospho-D-erythronate

71 L-glutamine

38 L-glutamate

72 L-aspartyl-[tRNAAsn]

73 L-asparaginyl-[tRNAAsn]

74 L-asparagine

41 L-aspartate

75 glucosamine 6-phosphate

76 N-acetylglucosamine 6-phosphate

77 N-acetylglucosamine 1-phosphate

58 UDP-N-acetyl-D-glucosamine

57 N-acetyl-D-mannosamine

78 N-acetyl-neuraminate

79 CMP-N-acetyl-neuraminate

80 D-mannopyranose 6-phosphate

81 a-D-mannose 1-phosphate

82 GDP-a-D-mannose

83 glycine

84 L-serine

85 tetrahydrofolate

86 5,10-methenyltetrahydrofolate

87 7,8-dihydrofolate

88 5,10-methylenetetrahydrofolate

89 N10-formyltetrahydrofolate

90 N-formyl-L-methionyl-[initiator tRNAmet]

91 L-methionyl-[initiator tRNAMet]

92 4a-hydroxy-N10-formyltetrahydrofolate

93 N10-formyl-7,8-dihydrofolate

94 6,7-dihydropteridine

95 4a-hydroxytetrahydropteridine

96 4a-hydroxy-L-erythro-5,6,7,8-tetrahydrobiopterin

97 (6R)-L-erythro-6,7-dihydrobiopterin

98 L-methionine

99 L-methionine-(R)-S-oxide

100 S-adenosyl-L-methionine

101 S-adenosyl-L-homocysteine

102 2-[(3S)-3-carboxy-3-(dimethylammonio)propyl]-L-histidine-[translation elongation factor 2]

103 2-[(3S)-3-amino-3-carboxypropyl]-L-histidine-[translation elongation factor 2]

104 2-[(3S)-3-carboxy-3-(methylammonio)propyl]-L-histidine-[translation elongation factor 2]

105 diphthine-[translation elongation factor 2]

106 diphthamide-[translation elongation factor 2]

107 S-methyl-5'-thioadenosine

108 glutathione

109 S-(2-hydroxyacyl)glutathione

110 2-hydroxy carboxylate

111 (R)-S-lactoylglutathione

26 (R)-lactate

112 protein with reduced L-cysteine residues

113 reduced glutaredoxin

114 oxidized glutaredoxin

115 ophthalmate

116 g-L-glutamyl-(S)-2-aminobutanoate

117 DL-allothreonine

118 acetaldehyde

119 [acetoin dehydrogenase E2 protein] N6-lipoyl-L-lysine

120 [acetoin dehydrogenase E2 protein] N6-dihydrolipoyl-L-lysine

121 acetoin

122 norophthalmate

123 g-L-glutamyl-D-alanine

124 hydrogen peroxide

125 oxygen

126 L-cysteine

127 [1-Cys-peroxiredoxin]-S-hydroxy-L-cysteine

128 oxidized thioredoxin

129 organic hydroperoxide

130 alcohol

131 [2-Cys peroxiredoxin] with hydroxylated L-cysteine

132 3'-phosphoadenylyl-sulfate

128 oxidized thioredoxin

133 reduced thioredoxin

134 [1-Cys-peroxiredoxin]-L-cysteine

127 [1-Cys-peroxiredoxin]-S-hydroxy-L-cysteine

98 L-methionine

99 L-methionine-(R)-S-oxide

135 protein-L-methionine-(S)-S-oxide

136 protein-L-methionine-(R)-S-oxide

137 [protein]-L-methionine

138 [2-Cys peroxiredoxin] with disulfide bond

139 [2-Cys peroxiredoxin] with reduced L-cysteine residues

131 [2-Cys peroxiredoxin] with hydroxylated L-cysteine

140 oxidized [peroxiredoxin disulfide reductase]

141 reduced [peroxiredoxin disulfide reductase]

142 reduced NrdH glutaredoxin-like protein

143 oxidized NrdH glutaredoxin-like protein

144 L-alanine

145 small subunit of molybdopterin synthase

146 carboxy-adenylated [small subunit of molybdopterin synthase]

147 6-(L-threonyl)-2-acetamido-2-deoxy-b-uronatemannosyl-(1->4)-2,3-diacetamido-2,3-dideoxy-b-D-uronateglucosyl-(1->3)-N-acetyl-a-D-glucosaminyl-(C55 o-saturated dolichyl phosphate)

148 o-saturated C55 dolichol phosphate

149 [M. voltae protein]-L-asparagine-[trisaccharide]

150 (1,4-a-D-glucosyl)(n+1) glycogenin

151 (1,4-a-D-glucosyl)(n) glycogenin

152 UDP-a-D-glucose

153 a-D-glucopyranose 1-phosphate

154 UDP-a-D-glucuronate

155 (R)-lipoate

156 [lipoyl-carrier protein]-N6-[(R)-lipoyl]-L-lysine

157 [(R)-lipoyl]adenylate

158 [glycine-cleavage complex H protein] N6-[(R)-lipoyl]-L-lysine

159 [glycine-cleavage complex H protein] N6-dihydrolipoyl-L-lysine

160 [glycine cleavage system lipoyl-carrier protein]-L-lysine

161 [lipoyl-carrier protein]-N6-[(R)-dihydrolipoyl]-L-lysine

162 [lipoyl-carrier protein]-L-lysine

163 (2E,6E)-farnesyl diphosphate

164 presqualene diphosphate

165 squalene

166 di-trans,octa-cis-undecaprenyl diphosphate

167 geranylgeranyl diphosphate

168 b-nicotinate D-ribonucleotide

169 nicotinate adenine dinucleotide

170 nicotinate

171 5-phospho-a-D-ribose 1-diphosphate

172 ATP

173 cyclic AMP
